# Diversity and antibacterial potential of the Actinobacteria associated with *Apis mellifera ligustica*

**DOI:** 10.3389/fmicb.2022.1056176

**Published:** 2022-12-16

**Authors:** Pu Cui, Haoyang Wu, Taoshan Jiang, Jian Tao, Zhiwei Zhu, Peng Liu, Linsheng Yu, Yinglao Zhang

**Affiliations:** ^1^School of Life Sciences, Anhui Agricultural University, Hefei, China; ^2^School of Plant Protection, Anhui Agricultural University, Hefei, China

**Keywords:** honeybee, Actinobacteria, antibacterial activity, secondary metabolites, community composition

## Abstract

Insect-associated Actinobacteria are a potentially rich source of novel natural products with antibacterial activity. Here, the community composition of Actinobacteria associated with *Apis mellifera ligustica* was investigated by integrated culture-dependent and independent methods. A total of 61 strains of *Streptomyces* genera were isolated from the honeycomb, larva, and different anatomical parts of the honeybee’s body using the culture-dependent method. Amplicon sequencing analyses revealed that the actinobacterial communities were dominated by the family of Bifidobacteriaceae and Microbacteriaceae in the honeybee gut, and Nocardiaceae and Pseudonocardiaceae in the honeycomb, whereas only *Streptomyces* genera were isolated by the culture-dependent method. Culture-independent analyses showed more diverse actinobacterial communities than those of culture-dependent methods. The antibacterial bioassay showed that most crude extracts of representative isolates exhibited antibacterial activities. Among them, the crude extract of *Streptomyces* sp. FCF01 showed the best antibacterial activities against *Staphylococcus aureus*, *Micrococcus tetragenus*, and *Pseudomonas syringae* pv. *actinidiae* (Psa) with the disc diameter of inhibition zone diameter (IZD) of 23.00, 15.00, and 13.33 mm, respectively. Chemical analysis of *Streptomyces* sp. FCF01 led to the isolation of three secondary metabolites, including mayamycin (**1**), mayamycin B (**2**), and N-(2-Hydroxyphenyl) acetamide (**3**). Among them, compound **1** displayed strong antibacterial activity against *S. aureus*, *M. tetragenus*, and Psa with minimum inhibitory concentrations (MIC) values of 6.25, 12.5, and 6.25 μg/ml, respectively. In addition, two novel derivative compounds **1a** and **1b** were synthesized by acetylation of compound **1**. Both compounds **1a** and **1b** displayed similar antibacterial activities with those of metabolite **1**. These results indicated that *Streptomyces* species associated with honeybees had great potential in finding antibiotics.

## Introduction

Bee-associated microorganisms play an important role in nutritional function, pathogen protection, host behavior regulation (Menezes et al., 2015; Paludo et al., 2018, 2019; Zheng et al., 2018; Paxton, 2020; Zhang et al., 2022). These microorganisms are not only sourced from the gut of bees, but also from other anatomical parts of bees, food sources (pollen, beebread, and honey), and honeycombs (Khan et al., 2020). Bacteria are common bee-associated microorganisms and have also been the focus of attention (Zheng et al., 2018). In contrast to Gram-negative bacteria, Gram-positive Actinobacteria associated with bees are less well studied (Promnuan et al., 2021). Bee-associated Actinobacteria have been isolated from diverse bee species, including honeybees (*Apis mellifera*, *A. cerana*, *A. florae*, *A. dorsata*, and *A. andreniformis*), stingless bees, and wasps and other key components of bees (including larvae, adults, brood cells, hive, pollen, beebread, honey, and honeycomb; Promnuan et al., 2009, 2020, 2021; Poulsen et al., 2011; Anjum et al., 2018; Cambronero-Heinrichs et al., 2019; Grubbs et al., 2021). Isolated Actinobacteria have mainly belonged to the genera *Streptomyces*, and some other rare genera, such as *Micromonospora*, *Nonomuraea*, *Nocardiopsis*, *Actinomadura*, and *Saccharopolyspora*. Furthermore, some bee-associated Actinobacteria have good antimicrobial potential against the pathogen of *Paenibacillus*, human pathogens, and plant-pathogenic bacteria (Cambronero-Heinrichs et al., 2019; Rodríguez-Hernández et al., 2019; Promnuan et al., 2021). Previous studies have found that bee-associated Actinobacteria produced antibiotics to inhibit pathogens of bees (Engl et al., 2018; Rodríguez-Hernández et al., 2019; Menegatti et al., 2020; Grubbs et al., 2021). Thus, bee-associated Actinobacteria harbor the biosynthetic potential to produce antimicrobial compounds. Although antibiotics have been found in some bee-associated Actinobacteria, they are still a huge and underexplored repository to search for novel antibiotics or natural products.

Honeybee (*A. mellifera ligustica*) is a kind of eusocial insect, which is widely distributed in primary beekeeping areas of China (Xiao et al., 2021). To the best of our knowledge, fewer studies have focused on Actinobacteria associated with *A. mellifera ligustica* compared with other bee species. In this study, we investigated the diversity of Actinobacteria from honeybee (*A. mellifera ligustica*) by using culture-dependent and independent approaches, and assessed the antibacterial activity of culturable Actinobacteria. Additionally, we described the isolation, structural elucidation, and derivatization of secondary metabolites produced by one *Streptomyces* strain with antibacterial activity.

## Materials and methods

### Sample collection

Honeybee samples (including 35 larvae, 49 adults, and honeycomb) were collected from the Institute of Apicultural Research, Anhui Agricultural University, Hefei, China (GPS: 31^°^53′ N, 117^°^20′ E) between November 2021 and April 2022. The honeybee larvae and adults starved for 24 h. Some honeybee samples were stored at −20°C for isolation and −80°C for DNA extraction, respectively.

### Actinobacteria isolation

Initially, seven adult honeybees were separately placed into 10 ml of sterile water in an autoclaved tube to obtain Actinobacteria from external isolation. Then, seven individuals of honeybee larvae and adults were separately placed in an autoclaved 50 ml tube with 10 ml 75% ethanol for 2 min (Xu et al., 2020), followed by rinsing in 10 ml of sterile water three times (30 s each). For the honeycomb, one gram sample was also processed using the same method. After external sterilization, sterile forceps were used to divide samples of the adult honeybee to get the head, gut, and abdomen. According to the earlier report (Chevrette et al., 2019), each body part of the adult honeybee, larvae, and honeycomb was fully homogenized separately in 10 ml of sterile water. Finally, the homogenates were diluted in a 10-fold series (i.e., 10^−1^, 10^−2^, 10^−3^), and an aliquot of 100 μl suspension was spread to six different Actinobacteria-selective media types (Supplementary Table S1), including cellulose-casamino acid (CC), starch casein agar (SCA), Reasoner’s 2A agar (R2A), Gause’s No. 1 (GS), modified HV (M-HV), and Actinobacteria isolation agar (AIA). All isolation media were amended with nystatin (50 mg/L), nalidixic acid (25 mg/L), cycloheximide (25 mg/L), and potassium dichromate (25 mg/L) to suppress the growth of Gram-negative bacteria and fungi (Li et al., 2021). The cultures were incubated at 28°C for 1–4 weeks. The actinobacterial colonies obtained after incubation were transferred onto Gause’s No.1 agar and then preserved on slants at 4°C or as glycerol suspensions (25%, v/v) at −80°C until use.

### Molecular identification and phylogenetic analysis of isolates

Isolates were cultivated on Gause’s No.1 medium at 28°C, and then preliminarily identified according to their distinct morphological characteristics. DNA extraction of each isolate was performed as described by Jiang et al. (2018). The specific primer pair 27F (5′-TCCTCCGCTTATTGATATGC-3′)/1492R (5′-GGTTACCTTGTTACG ACTT-3′) were used to amplify 16S rRNA based on the actinobacterial genomic DNA, and all PCR reactions were conducted according to the previous method (Long et al., 2022). Then, each successful product was sent to Tsingke Biotechnology Co., Ltd. (Beijing, China) for sequencing. All achieved sequences were compared with those of closely related reference strains and obtained the top hits (described species) with type material sequences using the EzTaxon-e server (Kim et al., 2012; https://www.ezbiocloud.net/). Neighbor-joining phylogenetic tree was constructed using the MEGA software version 5.0, and bootstrap replication (1,000 replications) was used to assess the topology of the phylogenetic tree (Felsenstein, 1985). The obtained gene sequences were deposited in the GenBank database under accession numbers OP491886-OP491954.

### Culture-independent community analysis

The external sterilization of seven adult honeybees and one gram honeycomb were the same as those mentioned above method to obtain the honeybee gut and honeycomb. The total community DNA of the honeybee samples was performed using the Fast DNA Extraction Kit referring to the manufacturer’s instructions. Then, the yield and purity of DNA were detected with electrophoresis on a 2% agarose gel. Each sample was repeated three times. The hypervariable regions V4 of the 16S rRNA gene were targeted for amplification by PCR with primers 515F and 806R. The PCR reaction was carried out with 15 μl of Phusion® High-Fidelity PCR Master Mix (New England Biolabs), 2 μM of forward/reverse primers, and about 10 ng of template DNA. The reaction conditions of PCR were performed as described method (Cui et al., 2022). Mixture PCR products were purified with Qiagen Gel Extraction Kit (Qiagen, Germany). The PCR products were pooled in an equimolar ratio and purified with Qiagen Gel Extraction Kit (Qiagen, Germany). The sequencing library was generated using TruSeq® DNA PCR-Free Sample Preparation Kit (Illumina, United States) according to the manufacturer’s instructions, and index codes were added. The library quality was evaluated on the Qubit@ 2.0 Fluorometer (Thermo Scientific) and Agilent Bioanalyzer 2,100 system. Finally, the library was sequenced on an Illumina NovaSeq platform using 250 bp paired-end reads.

Raw data obtained from sequencing were merged using FLASH (V1.2.7; Magoč and Steven, 2011). Then, quality filtering on the raw tags was performed to obtain high-quality clean tags according to the QIIME (V1.9.1; Caporaso et al., 2010). Subsequently, the clean tags were compared with the Silva database using UCHIME Algorithm to detect and remove chimera sequences (Edgar et al., 2011; Haas et al., 2011). The sequences with ≥97% pairwise identity were assigned to the same operational taxonomic units (OTUs) by Uparse software (Uparse v7.0.1001; Edgar, 2013). For each OTU, the Silva Database was used based on the Mothur algorithm to annotate taxonomic information (Quast et al., 2013). Raw data were available from the NCBI Short Read Archive under accession numbers PRJNA883759 and PRJNA882994.

### Extracts preparation and antibacterial assay

Based on morphological characteristics and molecular identification, 49 isolates were selected for small-scale fermentation to screen isolated actinobacterial strains with antibacterial activity. Strains were cultivated in a 250 ml Erlenmeyer flask containing 150 ml of Gause’s No.1 liquid medium and incubated at 28°C under 180 rpm for 7 days. The culture was passed through four layers of cheesecloth to get the supernatant. Then, the supernatant was extracted three times by using a separatory funnel with ethyl acetate (EtOAc, 1:1, v/v). The upper organic layer was condensed by a vacuum to obtain the crude extract for further experimental use.

The antibacterial activity of crude extracts of isolated strains was determined by using the filter paper disc method (Xu et al., 2020). Specifically, crude extracts were dissolved separately in acetone to get a concentration of 10 mg/ml. 5 μl of the tested crude extract was dripped on a sterile paper disk (diameter, 6 mm), then the paper disk was placed on the Luria broth (LB) agar plates containing the tested strains. Four bacterial strains including *Staphylococcus aureus* (ATCC6538), *Micrococcus tetragenus* (ATCC35098), *Escherichia coli* (ATCC8739), and *Pseudomonas syringae* pv. *actinidiae* (Psa) were used as indicator pathogens, three of which (*E. coli*, *M. tetragenus*, and *S. aureus*) were cultivated at 37°C for 24–36 h. Psa was cultivated at 28°C for 24–36 h. In addition, 5 μl of pure acetone alone and gentamicin sulfate with a concentration of 10 mg/ml served as the blank control and positive control, respectively. The plates were prepared in triplicate. Lastly, the diameters of inhibition zone diameter (IZD, in mm) were measured for evaluating antibacterial activity.

### Isolation and characterization of secondary metabolite

One strain FCF01 with the best antibacterial activity was selected for the purification and identification of compounds in this study. The strain FCF01 was inoculated into a 250 ml Erlenmeyer flask containing 150 ml of Gause’s No. 1 liquid medium and incubated at 28°C under 180 rpm for 3 days. Then, aliquots (15 ml) of the seed culture were transferred into 1,000 ml Erlenmeyer flasks filled with 400 ml of the same medium and cultured at 28°C for 7 days with shaking at 180 rpm. The fermentation broth (16 L) was filtered, and the supernatant was extracted with EtOAc (3 × 16 L). The EtOAc phase was concentrated by a rotary evaporator under reduced pressure to obtain 2.5 g of crude extract.

The crude extract was divided into six fractions using column fractionation packed with silica gel (200–300 mesh) eluting with dichloromethane (CH_2_Cl_2_)/methanol (MeOH; 100,0, 100,1, 100,2, 100:4, 100:8, and 100:16, v/v; fractions 1–6). Fraction 6 (CH_2_Cl_2_/MeOH, 100:16, v/v) was further fractionated on a silica gel column, eluting with CH_2_Cl_2_/MeOH (100,16, v/v) to yield compound **1** (310 mg) and subfraction (R1). The subfraction R1 was loaded onto a Sephadex LH-20 column (MeOH) to give compound **2** (1.6 mg). Fraction 3 (CH_2_Cl_2_/MeOH, 100:2, v/v) was loaded onto a Sephadex LH-20 column (MeOH) to give compound **3** (2 mg).

The structure of the secondary metabolites was determined by using spectroscopic analysis. NMR spectra were measured with Agilent 600 MHz DD2 spectrometers (Agilent, United States). HR-ESI-MS data were obtained by using a TripeTOF 4,600 mass analyzer (Bruker, United States).

### Acetylation of compound 1

According to the previous method with some modifications (Park et al., 2019), a solution of 0.075 mmol of compound **1** was added in 2.0 ml of dimethylformamide (DMF) and 21 μl of Acetic anhydride (Ac_2_O). After stirring the mixture for 4 h at 25°C, distilled water was added and the mixture was extracted with EtOAc (3 × 15 ml). The resulting mixture was concentrated *in vacuo* and purified by analytical HPLC (XBridge C18 column, 250 × 10 mm i.d., 5 μm, 1.0 ml/min, 0.0–30.0 min, and CH_3_OH: H_2_O = 90:10) to obtain compounds **1a** (t_R_ = 15.8 min, 6.0 mg) and **1b** (t_R_ = 18.2 min, 2.0 mg).

### Antibacterial activities of compounds

The antibacterial activity of the compounds was determined by the methods of minimum inhibitory concentrations (MICs; Li et al., 2014) and filter paper disc method (Xu et al., 2020). Four bacteria including *S. aureus*, *M. tetragenus*, *E. coli*, and Psa were used to assess the antibacterial activity. MICs of compounds were measured in disposable 96-well microtiter dishes. Specifically, a stock solution of each tested compound (200 μg/ml) was further 2-fold diluted in LB liquid medium and added separately into individual wells (100 μl/well) with a series of concentrations ranging from 100 to 3.13 μg/ml. Then, a 100 μl standard amount of the tested bacteria (1.0 × 10^6^ CFU/ml) were added per well. The 96-well plates were incubated at 37°C for 12–14 h. The control wells contained the same amount of culture broth and bacteria without the compound. The lowest concentration of compounds that inhibit bacterial growth was defined as MIC, as shown by no turbidity. Gentamicin sulfate was used as the positive control. Each test was performed three times. The diameters of IZD (in mm) of the compounds were determined by using the filter paper disc method as previously described for the antibacterial activity of crude extracts of isolated strains.

## Results

### Isolation and identification of Actinobacteria

**Figure 1:**
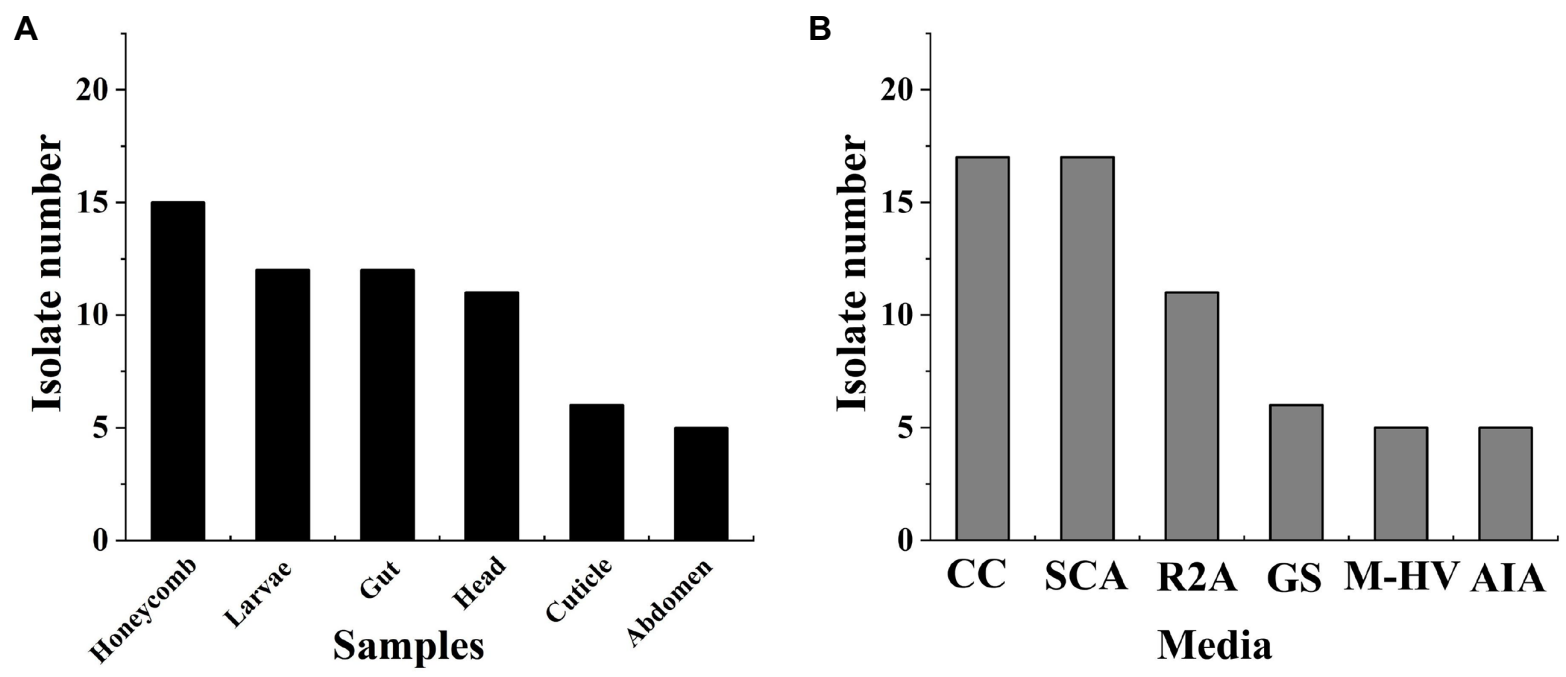
Statistics of Actinobacteria isolated from honeybee samples. **(A)** Different isolation parts of samples; **(B)** Different isolation media.

In this study, a total of 61 isolates were obtained from the honeycomb, larvae, and different parts of the adult honeybee on six different media. Among them, 15 isolates were isolated from the honeycomb, 12 from larvae, 12 from the honeybee gut, 11 from the honeybee head, six from the honeybee cuticle, and five from the honeybee abdomen (Figure 1A). The majority of isolates were recovered from CC (17 isolates, 27.9%) and SCA medium (17 isolates, 27.9%), followed by R2A (11 isolates, 18.0%), GS (six isolates, 9.8%), AIA (five isolates, 8.2%), and M-HV (five isolates, 8.2%; Figure 1B). Thus, the CC and SCA media favored the isolation of *Streptomycetes*.

All isolates were identified using 16S rRNA sequencing and analyzed by BLAST. The results showed that all isolates had high similarity to members of the genus *Streptomyces* belonging to the family Streptomycetaceae (Supplementary Table S2; Supplementary Figure S1). Especially, EzTaxon analysis of the 16S rRNA gene sequences revealed that some isolates showed relatively low similarities to the type strains of the corresponding genera. For example, two isolates (BTF01 and BTF07) showed only 98.67% similarity to *S. cavourensis* NBRC 13026^T^, which indicated a potential new species. One isolate BTF12 also showed similarity to *S. cavourensis* NBRC 13026^T^ with a low identity of 98.74%. Moreover, some similar actinobacterial strains were isolated from different parts of the honeybee, larva, and honeycomb. For instance, BTF27, BFF03, YCF15, and BCF05, which were isolated from the head, abdomen, larva, and gut, respectively, showed 99.86% similarity to *S. cavourensis* NBRC 13026^T^.

### Culture-independent community

**Figure 2:**
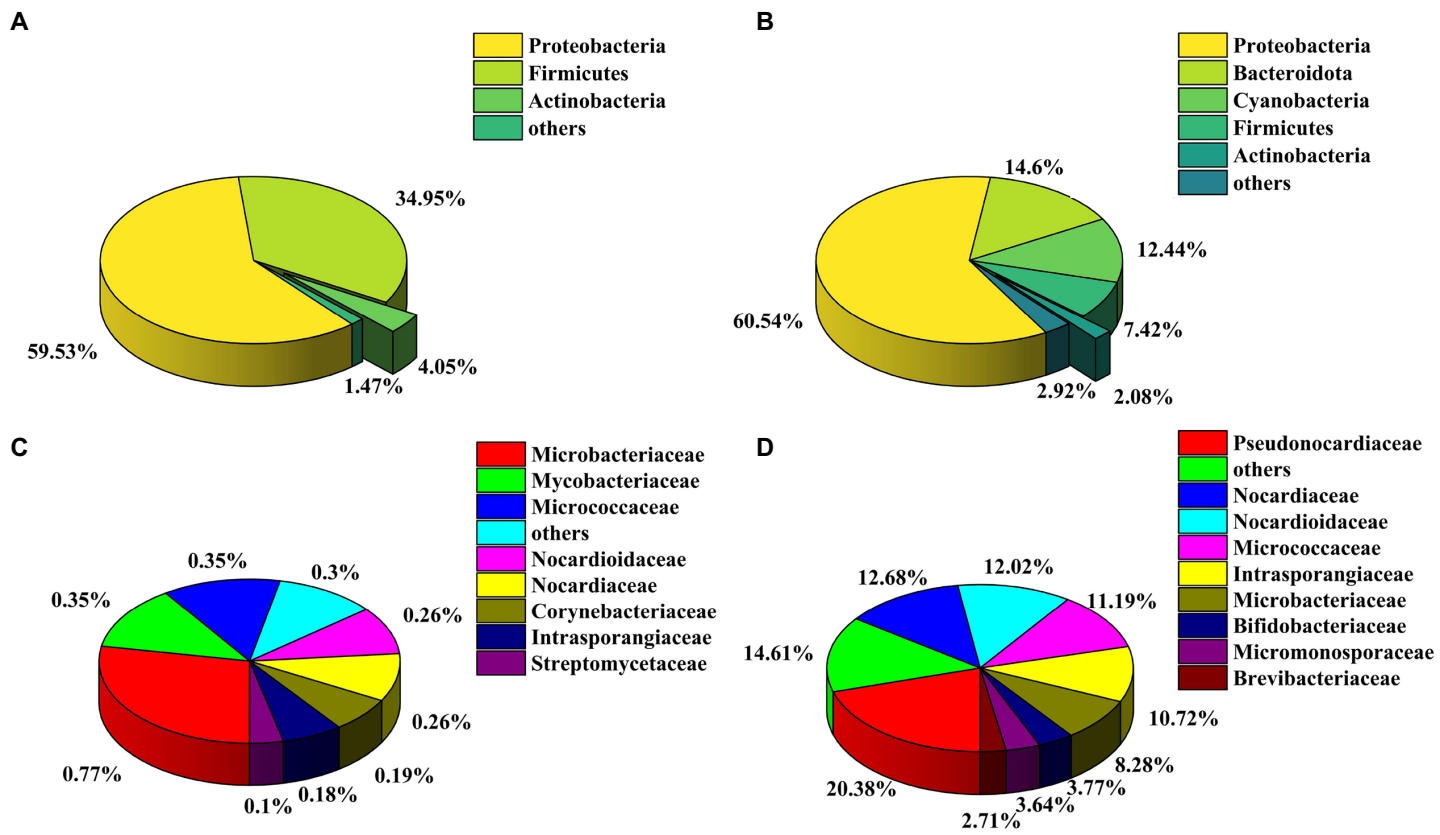
Analysis of culture-independent bacterial communities. Relative abundance of OTUs at the phylum level of honeybee gut **(A)** and honeycomb **(B)**; Relative abundance of OTUs at the family level from the phylum Actinobacteria of honeybee gut **(C)** and honeycomb **(D)**.

The bacterial communities in the honeybee gut and honeycomb were analyzed by sequencing the V4 region of the bacterial 16S rDNA gene. Amplicon sequencing yielded a total of 430,065 high-quality bacterial clean reads distributed across 1918 OTUs. According to taxonomic classifications of OTUs, a total of 29 known phyla were identified in the samples of honeybee gut, wherein the Proteobacteria (59.53%) was the most abundant phylum, followed by the phylum Firmicutes (34.95%) and Actinobacteria (4.05%; Figure 2A). Proteobacteria (60.54%) was also the dominant phylum in the honeycomb. However, Actinobacteria was the fifth most prevalent phylum in the honeycomb with a relative abundance of 2.08% (Figure 2B). The actinobacterial communities were further analyzed at the family level, in which 15 families were identified from the honeybee gut, and 23 families from the honeycomb (Supplementary Table S3, S4). Among them, Pseudonocardiaceae (20.38%), Nocardiaceae (12.68%), Nocardioidaceae (12.02%), Micrococcaceae (11.19%), and Intrasporangiaceae (10.72%) had higher abundance in the honeycomb (Figure 2D). However, the relative abundance of the family Bifidobacteriaceae in honeybee gut was very high (97.24%), followed by the family Microbacteriaceae (0.77%), Mycobacteriaceae (0.35%), and Micrococcaceae (0.35%; Figure 2C; Supplementary Table S3). In addition, the family Streptomycetaceae showed lower relative abundance in both the honeybee gut (0.10%) and honeycomb (0.26%).

### Screening for antibacterial activities

The antibacterial activities of crude extracts were performed by the filter paper disc method. The results showed that 38 of the 49 isolates (77.6%) exhibited antibacterial activities against at least one of the tested bacterial strains (Supplementary Table S5). Especially, three isolates (BTF05, YCF09, and BCF02) exhibited antibacterial activities against both Gram-positive and Gram-negative bacteria. FCF01 and BFF04 showed moderate to excellent antibacterial activities against *S. aureus* with an IZD of more than 12.00 mm, which was slightly weaker than the positive gentamicin sulfate with an IZD of 21.67 mm. BTF05, BTF15, and BCF02 exhibited remarkable inhibitory activities against *M. tetragenus* with an IZD of more than 25.00 mm, which was slightly weaker than the positive gentamicin sulfate with an IZD of 37.67 mm. Furthermore, the strain FCF01 exhibited moderate antibacterial activity against *M. tetragenus* with an IZD of 15.00 mm. In addition, eight and 20 isolates exhibited antibacterial activities against *E. coli* and Psa, respectively.

### Identification of secondary metabolites and derivative compounds

**Figure 3:**
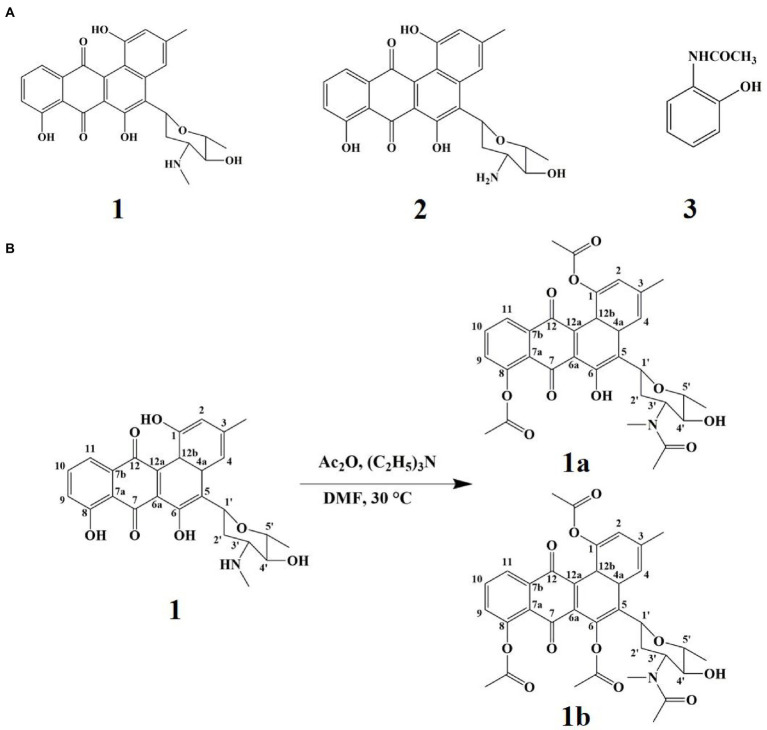
The secondary metabolites and derivative compounds of strain FCF01. **(A)** The structure of compounds 1–3; **(B)** Derivatization pathways of compound 1.

Three compounds were purified from Gause’s No. 1 liquid fermentation product of *Streptomyces* sp. FCF01 and their structures were determined to be mayamycin (**1**; Bo et al., 2018), mayamycin B (**2**; Bo et al., 2018), and N-(2-Hydroxyphenyl) acetamide (**3**; Shang et al., 2012; Figure 3A) by spectroscopic data analyses and comparison of their data in the literature. The synthesis pathways of derivative compounds based on compound **1** are shown in Figure 3B. The structures of derivatives (**1a** and **1b**) were identified based on the 2D-NMR spectroscopic analysis and (HR)-ESI-MS data.

Mayamycin (**1**): brown solid; HR-ESI-MS: *m/z*: 464.1677 [M + H]^+^, calculated for C_26_H_25_NO_7_ 463.1631; ^1^H NMR (600 MHz, CD_3_OD) *δ*: 8.00 (1H, s, 4-H), 7.75 (1H, td 7.62, 10-H), 7.58 (1H, s, 11-H), 7.29 (1H, s, 9-H), 6.74 (1H, s, 2-H), 5.73(1H, d 11.04, 1´-H), 3.61 (1H, m, 5´-H), 3.52 (1H, td 9.48, 4´-H), 3.43 (1H, m, 3´-H), 2.75 (3H, s, 3′-N-CH_3_), 2.48 (3H, s, 3-CH_3_), 2.35 (2H, d 12.36, 2´-H), 1.47 (3H, d 5.94, 5´-CH_3_); ^13^C NMR (150 MHz, CD_3_OD): 194.2 (C7), 188.0 (C12), 162.9 (C8), 156.6 (C1), 143.5 (C3), 139.9 (C4a), 138.9 (C6a), 137.8 (C11a), 124.8 (C9), 120.3 (C11), 119.4 (C12a), 117.8 (C12b), 117.4 (C4), 116.4 (C7a), 114.7 (C2), 79.2 (C5´), 74.2 (C4´), 72.8 (C1´), 62.9 (C3´), 32.5 (C2´), 31.1 (3′-N-CH_3_), 22.64 (3-CH_3_), and 18.6 (C5´-CH_3_).

Mayamycin B (**2**): brown solid; HR-ESI-MS: *m/z*: 450.1550 [M + H]^+^, calculated for C_25_H_23_NO_7_ 449.1475; ^1^H NMR (600 MHz, CD_3_OD) *δ*: 7.99 (1H, s, 4-H), 7.75 (1H, td 7.92, 10-H), 7.58 (1H, d 7.38, 11-H), 7.28 (1H, d 8.46, 9-H), 6.74 (1H, s, 2-H), 5.71(1H, d 11.7, 1´-H), 3.58 (1H, m, 5´-H), 3.43 (1H, m, 4´-H), 3.43 (1H, m, 3´-H), 2.52 (1H, m, 2´-H), 2.45 (3H, s, 3-CH_3_), 2.20 (1H, d 13.08, 2´-H), and 1.43 (3H, d 6.12, 5´-CH_3_).

N-(2-Hydroxyphenyl) acetamide (**3**): white powder; HR-ESI-MS: *m/z*: 152.0708 [M + H]^+^, calculated for C_8_H_9_NO_2_ 151.0633; ^1^H NMR (600 MHz, acetone-*d_6_*) *δ*: 9.38 (1H, s, 1-NH), 9.26 (1H, s, 2-OH), 7.37 (1H, d 7.92, 6-H), 7.02 (1H, td 7.98, 4-H), 6.89 (1H, d 7.86, 3-H), 6.79 (1H, td 7.86, 5-H), 2.20 (3H, s, NHAc); ^13^C NMR (150 MHz, acetone-*d_6_*): 171.2 (C7), 149.5 (C2), 127.8 (C1), 126.7 (C4), 122.9 (C6), 120.5 (C5), 119.1 (C3), and 23.5 (NHAc, CH_3_).

Compound **1a** was obtained as red powder, and its molecular formula C_32_H_31_NO_10_ was deduced from HR-ESI-MS data (*m/z*: 590.2018 [M + H]^+^ and 612.1837 [M + Na]^+^, calculated for C_32_H_32_NO_10_ 590.2027 and C_32_H_31_NO_10_Na 612.1846, respectively). The structure of compound **1a** was established through comparison with compound **1** and the detailed NMR data analysis of 2D-NMR (Supplementary Figures S2–S8). The ^1^H NMR spectrum of **1a** exhibited the presence of three acetyl groups at *δ*_H_ 2.18 (s, 3H), 2.30 (s, 3H), and 2.47 (s, 3H), respectively. ^13^C NMR (Table 1) and DEPT spectrum displayed 32 carbon resonances that were grouped into 16 aromatic carbons, 2 carbonyl carbons signal (*δ*_C_ 184.8, 188.5), 3 methyl groups carbons signal (*δ*_C_ 18.7, 22.8, and 31.3), 3 acetyl groups carbons signal (*δ*_C_ 21.2, 21.3, 22.6, 168.5, 169.5, 173.9), and 1 glycosyl carbon signal (*δ*_C_ 33.7, 57.1, 72.6, 73.8, 79.2). The HMBC correlations from 3′-NCOCH_3_ (*δ*_H_ 2.18) to 3′-NCOCH_3_ (*δ*_C_ 173.9, 22.6), from 1-OCOCH_3_ (*δ*_H_ 2.30) to 1-OCOCH_3_ (*δ*_C_ 168.5, 21.2) and C-1 (*δ*_C_ 147.5), and from 8-OCOCH_3_ (*δ*_H_ 2.47) to 8-OCOCH_3_ (*δ*_C_ 169.5, 21.3) and C-8 (*δ*_C_ 150.3) indicated the location of the three acetyl groups.

**Table 1:** ^1^H NMR and ^13^C NMR data of compounds 1a and 1b in CDCl_3_.

	**1a**		**1b**	
Position	*δ*_C_, mult.	*δ*_H_ (*J* in Hz)	*δ*_C_, mult.	*δ*_H_ (*J* in Hz)
1	147.5		147.6	
1-OCOCH_3_	168.5, 21.2	2.30	168.3, 21.2	2.33
2	123.0	7.14	125.1	7.35
3	140.7		140.8	
3-CH_3_	22.8	2.54	22.6	2.59
4	122.3	8.41	125.3	8.26
4a	138.4		136.8	
5	126.9		126.4	
6	154.1		149.7	
6-OH		12.61		
6-OCOCH_3_			169.7, 21.5	2.50
6a	119.1		120.5	
7	188.5		180.6	
7a	124.1		125.3	
8	150.3		149.7	
8-OCOCH_3_	169.5, 21.3	2.47	169.3, 21.2	2.44
9	129.4	7.40 (d, *J* = 7.92)	129.3	7.38 (d, *J* = 7.98)
10	136.2	7.81 (td, *J* = 7.68)	134.9	7.75 (td, *J* = 7.80)
11	124.4	7.95 (d, *J* = 7.44)	123.8	7.91 (d, *J* = 7.62)
11a	137.6		136.1	
12	184.8		185.3	
12a	134.8		134.3	
12b	118.0		120.5	
1′	72.6	5.71 (d, *J* = 10.2)	73.7	5.41
2′	33.7	1.90	34.0	1.25 (td, *J* = 7.32)
3′	57.1	4.91	56.8	4.86
3′-N-CH_3_	31.3	2.98	31.2	2.97
3′-NCOCH_3_	173.9, 22.6	2.18	174.0, 22.6	2.18
4′	73.8	3.46	73.7	3.42 (td, *J* = 9.48)
5′	79.2	3.66	79.3	3.60
5′-CH_3_	18.7	1.47 (d, *J* = 5.40)	18.6	1.44 (d, *J* = 5.70)

Compound **1b** was obtained as yellow powder, and its molecular formula C_34_H_33_NO_11_ was deduced from HR-ESI-MS data (*m/z*: 632.2133 [M + H]^+^ and 654.1946 [M + Na]^+^, calculated for C_34_H_34_NO_11_, 632.2121, and C_34_H_33_NO_11_Na 654.1952, respectively). The structure of compound **1b** was established through comparison with compound **1** and the detailed NMR data analysis of 2D-NMR (Supplementary Figures S9–S15). The ^1^H NMR (Table 1) spectrum of **1b** presented four acetyl groups signal *δ*_H_ 2.18 (s, 3H), 2.33 (s, 3H), 2.44 (s, 3H), and 2.50 (s, 3H). The ^13^C NMR and DEPT spectrum exhibited 34 carbon resonances including 16 aromatic carbons, 2 carbonyl carbon signal (*δ*_C_ 180.6, 185.3), 3 methyl groups carbons signal (*δ*_C_ 18.6, 22.6, 31.2), 4 acetyl groups carbons signal (*δ*_C_ 21.2, 21.2, 21.5, 22.6, 168.3, 169.3, 169.7, 174.0), and 1 glycosyl carbon signal (*δ*_C_ 34.0, 56.8, 73.7, 73.7, 79.3). The HMBC correlations from 3′-NCOCH_3_ (*δ*_H_ 2.18) to 3′-NCOCH_3_ (*δ*_C_ 174.0, 22.6), from 1-OCOCH_3_ (*δ*_H_ 2.33) to 1-OCOCH_3_ (*δ*_C_ 168.3, 21.2) and C-1 (*δ*_C_ 147.6), from 8-OCOCH_3_ (*δ*_H_ 2.44) to 8-OCOCH_3_ (*δ*_C_ 169.3, 21.2) and C-8 (*δ*_C_ 149.7), and from 6-OCOCH_3_ (*δ*_H_ 2.50) to 6-OCOCH_3_ (*δ*_C_ 169.7,21.5) and C-6 (*δ*_C_ 149.7) indicated the location of the four acetyl groups.

### Antibacterial activities of compounds

The MIC values and IZD of four compounds (**1**, **1a**, **1b**, and **3**) against different bacteria are presented in Table 2. The results showed that compound **1** exhibited strong antibacterial activities against *S. aureus*, *M. tetragenus*, and Psa in the MIC tests with the MIC values of 6.25, 12.50, and 6.25 μg/ml, which were comparable to those of positive gentamycin sulfate with the MIC values of 6.25, 12.50, and 3.13 μg/ml, respectively. In the filter paper disc tests, compound **1** also presented strong antibacterial activities against *S. aureus*, *M. tetragenus*, and Psa with the IZD of 16.33, 30.00, and 15.00 mm, which were slightly weaker than those of positive control with the IZD of 18.00, 36.33, and 19.67 mm, respectively. Compound **1a** displayed potent inhibitory activities against *S. aureus*, *M. tetragenus*, and Psa with MIC values of 12.50, 12.50, and 6.25 μg/ml, and the IZD of 15.00, 27.67, and 10.00 mm, respectively. Similarly, compound **1b** also showed potent inhibitory activities against *S. aureus*, *M. tetragenus*, and Psa with MIC values of 25, 12.50, and 12.50 μg/ml and the IZD of 14.67, 22.00, and 9.00 mm, respectively. Compound **3** displayed moderate antibacterial activities against *S. aureus*, *M. tetragenus*, and Psa with MIC values of 25, 25, and 12.5 μg/ml and the IZD of 8.33, 12.00, and 10.00 mm, respectively. However, the remaining *E. coli* was not susceptible to all compounds.

**Table 2:** Minimum inhibitory concentration (MIC) values (μg/mL) and inhibition zone diameter (IZD, mm) of compounds against four tested bacteria.

Compounds	*S. aureus*		*M. tetragenus*		*E. coli*		Psa	
	MIC	IZD^a^	MIC	IZD^a^	MIC	IZD^a^	MIC	IZD^a^
**1**	6.25	16.33 ± 0.47	12.5	30.00 ± 0.00	>100	NI	6.25	15.00 ± 0.00
**1a**	12.5	15.00 ± 0.00	12.5	27.67 ± 0.47	>100	NI	6.25	10.00 ± 0.00
**1b**	25	14.67 ± 0.47	12.5	22.00 ± 0.00	>100	NI	12.5	9.00 ± 0.00
**3**	25	8.33 ± 0.47	25	12.00 ± 0.00	>100	NI	12.5	10.00 ± 0.00
PC^b^	6.25	18.00 ± 0.00	12.5	36.33 ± 0.47	12.5	18.33 ± 0.47	3.13	19.67 ± 0.47

a Results are presented as the mean ± standard; “NI” means not inhibited; the concentration for the test is 30 μg/filter paper.

b Gentamycin sulfate as the positive control.

## Discussion

Actinobacteria, especially of the genus *Streptomyces*, has been one of the most essential sources for the discovery of antibiotics (Genilloud, 2017). Due to the continuing development of antibiotic resistance and the discovery of new antibiotics decreases, researchers were starting to search for *Streptomycetes* in other habitats rather than soil, such as insects, and plants (Jose et al., 2021). Compared to soil and plant-associated Actinobacteria, insect-associated Actinobacteria showed significant antimicrobial activity (Chevrette et al., 2019). Furthermore, insect-associated Actinobacteria have been a significant source of new microbial resources and novel natural products (Promnuan et al., 2011; Beemelmanns et al., 2017; Wang et al., 2020; Zhang et al., 2020). Here, 61 Actinobacteria, including two potential new species, were isolated and identified by culture-dependent and molecular biological methods. A 16S rRNA gene sequence similarity of 98.7% was considered a threshold value for species delimitation (Chun et al., 2018). The strains BTF01 and BTF07 showed less than 98.7% similarity to the closest species and thus were considered as potential new species. Meanwhile, the community composition of the honeybee gut and honeycomb was further analyzed by the culture-independent method. Moreover, three compounds and two novel derivative compounds, which had good antibacterial activities, were purified and characterized from *Streptomyces* sp. FCF01. Therefore, *Streptomyces* species associated with honeybees have great potential in finding new antibiotics.

To obtain extensive Actinobacteria from honeybee samples, we used six different types of isolation media, which have been found effective in the isolation of Actinobacteria. Among them, the CC and SCA media were the most effective as regards the number of obtained isolates. Both media have been also used to isolate rare Actinobacteria from caves and soils (Fang et al., 2017; Li et al., 2021). Chitin agar (CA) and ISP2 media were also widely used for the isolation of insect-associated Actinobacteria (Rodríguez-Hernández et al., 2019; Menegatti et al., 2020; Grubbs et al., 2021; Ortega et al., 2021). Therefore, these media can be further considered for the isolation of honeybee-associated Actinobacteria.

Actinobacteria isolated from the honeycomb, larvae, and different parts of adult honeybees (gut, head, cuticle, and abdomen) were investigated in this study. The result showed that *Streptomyces* was the predominant genus, which was consistent with other reports (Cambronero-Heinrichs et al., 2019; Grubbs et al., 2021). *Streptomyces* associated with bees might be the strains collected by many bees through pollen (Kim et al., 2019). Previous studies have focused on the isolation of Actinobacteria from the honeybee gut, honeycomb, pupae, pollen, and honey (Promnuan et al., 2009; Khan et al., 2017; Grubbs et al., 2021). However, the isolation of Actinobacteria from different parts of *A. mellifera* was neglected, such as the head and abdomen. Moreover, Poulsen et al. found the potential role of *Streptomyces* isolated from different parts of the wasp as antibiotic-producing symbionts (Poulsen et al., 2011). *A. mellifera* has been emerging as a potential source of novel species of Actinobacteria (Promnuan et al., 2011). In this study, two potentially new species were isolated from honeybee head. Considering the limitations of isolation methods, the culture-independent method was used to evaluate the actinobacterial community composition of insects in recent years (Wang et al., 2020).

Actinobacterial community structure was analyzed in both honeybee gut and honeycomb using the culture-independent method in this study. The phylum Actinobacteria was detected in the honeybee gut and honeycomb at 4.05 and 2.08% relative abundance respectively, which was a similarity to the results of the previous study (Liu et al., 2021). Fifteen and twenty-three actinobacterial families were detected by culture-independent method from the honeybee gut and honeycomb, respectively. However, only the family Streptomycetaceae was isolated, and some rare actinobacterial families, for instance, Nocardiaceae, Nocardioidaceae, Micrococcaceae, etc., were not detected by the culture-dependent method. A greater diversity of actinobacterial communities was detected using the culture-independent method compared to those of the culture-dependent method. This result provided the impetus to continue developing cultivation methods and strategies to culture rare Actinobacteria in future studies. For example, the treatment of samples and organism-media pairings could increase the recovery of rare Actinobacteria (Subramani and Aalbersberg, 2013; Oberhardt et al., 2015). Combined methods encompassing culture-dependent and independent techniques to retrieve broader actinobacterial communities have been used for different sources of the samples, such as dung beetle, desert sandy soils, and soybean (Liu et al., 2019; Kim et al., 2021; Li et al., 2021). Therefore, it was critical to use a combination of culture-dependent and independent methods to accurately assess the composition of the actinobacterial communities.

To validate that honeybee-associated Actinobacteria have antibacterial activity against pathogenic bacteria, 49 isolates were conducted antibacterial assay using three different human food-borne bacteria and one plant pathogenic bacterium. The results revealed that a high proportion of strains (77.6%) had antibacterial activities. There was also evidence that honeybee-associated Actinobacteria had potent antimicrobial activity against pathogens, including human food-borne bacteria (*S. aureus*), insect pathogen (*Beauveria bassiana*, *P. larvae*), plant pathogenic bacteria (*Ralstonia solanacearum*, *Xanthomonas campestris* pv. *Campestris*), and plant fungal pathogen (*Fusarium oxysporum*; *Botrytis cinerea*; Promnuan et al., 2020; Grubbs et al., 2021; Santos-Beneit et al., 2022). Actinobacteria associated with other insects had also been reported to have good antibacterial activities, such as termites, ants, and beetle (Scott et al., 2008; Wang et al., 2020; Long et al., 2022). Furthermore, some insect-associated Actinobacteria could produce substances with antibacterial activity (Zhang et al., 2020; Zhou et al., 2021).

Many antimicrobials with unique structures were identified from honeybee-associated Actinobacteria (Rodríguez-Hernández et al., 2019; Grubbs et al., 2021; Santos-Beneit et al., 2022). We investigated the secondary metabolites from one *Streptomyces* strain FCF01 with good antibacterial activity, which resulted in the isolation of mayamycin (**1**), mayamycin B (**2**), and N-(2-Hydroxyphenyl) acetamide (**3**). Among them, antibacterial compounds **1** and **2** have been reported to be produced by *Streptomyces* species and showed activity against *S. aureus* with the same MIC value of 64 μM (Bo et al., 2018; Alam et al., 2022). Furthermore, two novel derivatives (**1a** and **1b**) were further identified by acetylation of compound **1**. However, their antibacterial activities were slightly weaker than those of compound **1**, which indicated that the hydroxyl group of metabolite **1** might play a vital role in antibacterial activity. A similar study has shown the replacement of the phenolic hydroxyl group by aldehyde groups of the 15-copaenol resulted in weaker antibacterial activity (Espinoza et al., 2019).

## Conclusion

Here, the actinobacterial diversity of the honeybee samples was analyzed using both culture-dependent and independent methods. The results demonstrated the honeybee-derived sample harbored an excellent source of culturable actinobacterial strains. Antibacterial activity assays showed that most of these honeybee-associated Actinobacteria exhibited antibacterial activities. In addition, three known metabolites were purified from *Streptomyces* sp. FCF01 and two novel derivatives were identified by acetylation of compound **1**. Both compound **1** and its novel derivatives displayed potent antibacterial activity. These results suggest that honeybee-associated Actinobacteria represent a promising and underexplored resource for exploring antibiotics.

## Data availability statement

The datasets presented in this study can be found in online repositories. The names of the repository/repositories and accession number(s) can be found at: https://www.ncbi.nlm.nih.gov/, OP491886-OP491954 https://www.ncbi.nlm.nih.gov/, PRJNA883759 https://www.ncbi.nlm.nih.gov/, PRJNA882994.

## Author contributions

PC, HW, TJ, JT, ZZ, PL, and LY performed the experiments and analyzed the data. PC wrote the manuscript. YZ designed the experiments and reviewed the manuscript. All authors contributed to the article and approved the submitted version.

## Funding

This work was supported by the National Natural Science Foundation of China (NSFC; 32270015 and 32011540382), Anhui Province Natural Science Funds for Distinguished Young Scholars (2108085J18), and the University Graduate Science Research Project of Anhui Province (YJS20210200).

## Conflict of interest

The authors declare that the research was conducted in the absence of any commercial or financial relationships that could be construed as a potential conflict of interest

## Publisher’s note

All claims expressed in this article are solely those of the authors and do not necessarily represent those of their affiliated organizations, or those of the publisher, the editors and the reviewers. Any product that may be evaluated in this article, or claim that may be made by its manufacturer, is not guaranteed or endorsed by the publisher.
