# Supplementary material for: Diversity and antibacterial potential of the Actinobacteria associated with Apis mellifera ligustica
Source: Front Microbiol. 2022 Dec 16;13:1056176. doi: 10.3389/fmicb.2022.1056176 (PMC9800615; doi:10.3389/fmicb.2022.1056176)
Supplement: Supplementary file 1 [file Data_Sheet_1.docx]

Diversity and Antibacterial Potential of the Actinobacteria Associated with *Apis mellifera ligustica*

Pu Cui^1^, Haoyang Wu^1^, Taoshan Jiang^1^, Jian Tao^1^, Zhiwei Zhu^2^, Peng Liu^2^, Linsheng Yu^2^ and Yinglao Zhang^1^^*^

^1^School of Life Sciences, Anhui Agricultural University, Hefei 230036, China

^2^School of Plant Protection, Anhui Agricultural University, Hefei 230036, China

^*^Correspondence:

Yinglao Zhang

zhangyl@ahau.edu.cn;

**Supplementary Material**

**Table S1.** Media used for isolation and cultivation in this study.

**Table S2.** Taxonomic distribution of honeybee-associated actinobacteria and isolation media.

**Table S3.** The relative abundance of OTUs at family level from the phylum Actinobacteria of honeybee gut.

**Table S4**. The relative abundance of OTUs at family level from the phylum Actinobacteria of honeycomb.

**Table S5.** Antibacterial activity of honeybee-associated actinobacteria against the test pathogens (mm).

**Figure S1.** Neighbor-joining phylogenetic tree of 16S rRNA gene sequences from honeybee-associated actinobacteria and their phylogenetic neighbors. Numbers at nodes are bootstrap values (percentages of 1000 replications); only values >50% are shown.

**Figure S2.** The ^1^H NMR spectrum of compound **1a** in CDCl_3_ (600 MHz).

**Figure S3.** The ^13^C NMR spectrum of compound **1a** in CDCl_3_ (150 MHz).

**Figure S4.** The DEPT spectrum of compound **1a** in CDCl_3_ (150 MHz).

**Figure S5.** The ^1^H-^1^H COSY spectrum of compound **1a** in CDCl_3_ (600 MHz).

**Figure S6.** The HSQC spectrum of compound **1a** in CDCl_3_ (600 MHz).

**Figure S7.** The HMBC spectrum of compound **1a** in CDCl_3_ (600 MHz).

**Figure S8.** The ESI^+^ spectrum of compound **1a**.

**Figure S9.** The ^1^H NMR spectrum of compound **1b** in CDCl_3_ (600 MHz).

**Figure S10.** The ^13^C NMR spectrum of compound **1b** in CDCl_3_ (150 MHz).

**Figure S11.** The DEPT spectrum of compound **1b** in CDCl_3_ (150 MHz).

**Figure S12.** The ^1^H-^1^H COSY spectrum of compound **1b** in CDCl_3_ (600 MHz).

**Figure S13.** The HSQC spectrum of compound **1b** in CDCl_3_ (600 MHz).

**Figure S14.** The HMBC spectrum of compound **1b** in CDCl_3_ (600 MHz).

**Figure S15.** The ESI^+^ spectrum of compound **1b**.

**Table S1.** Media used for isolation and cultivation in this study.

| **Usage** | **Media** | **Components** |
| --- | --- | --- |
| Isolation | CC (cellulose-casamino acid) | Microcrystalline cellulose 1.0 g, casein amino acids 1.0 g, KNO_3_ 0.2 g, Na_2_HPO_4_ 0.5 g, MgSO_4_·7H_2_O 0.05 g, FeSO_4_·7H_2_O 0.01 g, agar 18 g, H_2_O 1000 mL, pH 7.2 |
|  | SCA (starch casein agar) | Soluble starch 10.0 g, casein 0.3 g, KNO_3_ 2.0 g, NaCl 2.0 g, K_2_HPO_4_ 2.0 g, MgSO_4_·7H_2_O 0.05 g, CaCO_3_ 0.02 g, FeSO_4_·7H_2_O 0.01 g, Vitamins (0.5 mg each of thiamine-HCl, riboflavin, niacin, pyridoxin-HCl, inositol, Ca-pantothenat, p-aminobenzoic acid, and 0.25 mg of biotin), agar 18.0 g, H_2_O 1000 mL, pH 7.5 |
|  | R2A (Reasoner’s 2A agar) | Yeast extract 0.5 g, peptone 0.5 g, casamino acid 0.5 g, glucose 0.5 g, soluble starch 0.5 g, K_2_HPO_4_ 0.3 g, MgSO_4_·7H_2_O 0.05 g, Na-pyruvate 0.3 g, Vitamins (0.5 mg each of thiamine-HCl, riboflavin, niacin, pyridoxin-HCl, inositol, Ca-pantothenat, p-aminobenzoic acid, and 0.25 mg of biotin), agar 18.0 g, H_2_O 1000 mL, pH 7.5 |
|  | GS (Gause’s No. 1) | Soluble starch 20 g, KNO_3_ 1.0 g, K_2_HPO_4_·3H_2_O 0.5 g, MgSO_4_·7H_2_O 0.5 g, NaCl 0.5 g, FeSO_4_·7H_2_O 0.01 g, agar 18 g, H_2_O 1000 mL, pH 7.4-7.6 |
|  | M-HV (modified HV medium) | Soluble starch 2.0 g, KNO_3_ 0.5 g, KCl 1.71 g, Na_2_HPO_4_ 0.5 g, CaCO_3_ 0.02 g, MgSO_4_·7H_2_O 0.05 g, FeSO_4_·7H_2_O 0.01 g, HV Multi-Vitamins ( thiamine 0.05 g, riboflavin 0.05 g, inose 0.05 g, pantothenic acid 0.05 g, p-aminobenzoic acid 0.05 g, vitamin B6 0.05 g, biotin 0.025 g, niacin 0.05 g, H_2_O 100 mL), agar 18.0 g, H_2_O 1000 mL |
|  | AIA (actinobacteria isolation agar) | Sodium propionate 4.0 g, sodium caseinate 2.0 g, K_2_HPO_4_ 0.5 g, L-Asparagine 0.1 g, MgSO_4_·7H_2_O 0.1 g, FeSO_4_·7H_2_O 0.001 g, Vitamins (0.5 mg each of thiamine-HCl, riboflavin, niacin, pyridoxin-HCl, inositol, Ca-pantothenat, p-aminobenzoic acid, and 0.25 mg of biotin), agar 18.0 g, H_2_O 1000 mL, pH 8.1 |
| Cultivation | GS (Gause’s No. 1) | Soluble starch 20 g, KNO_3_ 1.0 g, K_2_HPO_4_·3H_2_O 0.5 g, MgSO_4_·7H_2_O 0.5 g, NaCl 0.5 g, FeSO_4_·7H_2_O 0.01 g, agar 18 g, H_2_O 1000 mL, pH 7.4-7.6 |
|  | LB (Luria Bertani) | Yeast extract 5.0 g, NaCl 10.0 g, peptone 10.0 g, agar 18.0 g, H_2_O 1000 mL |

**Table S2.** Taxonomic distribution of honeybee-associated actinobacteria and isolation media.

| **Isolate** | **Host** | **Part** | **Closest Type Strain and Similarity** | **Coverage /Similarity** | **Medium** | **GenBank accession number** |
| --- | --- | --- | --- | --- | --- | --- |
| BTF01 | Honeybee | Head | *Streptomyces cavourensis* NBRC 13026 | 99.9/98.67 | GS | OP491886 |
| BTF07 | Honeybee | Head | *Streptomyces cavourensis* NBRC 13026 | 99.9/98.67 | GS | OP491887 |
| BTF12 | Honeybee | Head | *Streptomyces cavourensis* NBRC 13026 | 99.9/98.74 | SCA | OP491888 |
| FCF02 | Honeycomb |  | *Streptomyces cavourensis* NBRC 13026 | 99.9/98.81 | GS | OP491889 |
| FCF03 | Honeycomb |  | *Streptomyces cavourensis* NBRC 13026 | 99.9/98.81 | GS | OP491890 |
| BCF01 | Honeybee | Gut | *Streptomyces cavourensis* NBRC 13026 | 99.9/98.88 | GS | OP491892 |
| FCF23 | Honeycomb |  | *Streptomyces cavourensis* NBRC 13026 | 99.9/99.09 | SCA | OP491893 |
| BTF08 | Honeybee | Head | *Streptomyces cavourensis* NBRC 13026 | 99.9/99.15 | GS | OP491896 |
| FCF40 | Honeycomb |  | *Streptomyces cavourensis* NBRC 13026 | 99.9/99.23 | R2A | OP491898 |
| FCF44 | Honeycomb |  | *Streptomyces cavourensis* NBRC 13026 | 99.9/99.23 | R2A | OP491899 |
| BFF05 | Honeybee | Abdomen | *Streptomyces cavourensis* NBRC 13026 | 99.9/99.23 | CC | OP491900 |
| FCF30 | Honeycomb |  | *Streptomyces cavourensis* NBRC 13026 | 99.9/99.30 | AIA | OP491901 |
| BBF11 | Honeybee | Abdomen | *Streptomyces cavourensis* NBRC 13026 | 99.9/99.30 | CC | OP491902 |
| FCF43 | Honeycomb |  | *Streptomyces cavourensis* NBRC 13026 | 99.9/99.37 | CC | OP491904 |
| FCF47 | Honeycomb |  | *Streptomyces cavourensis* NBRC 13026 | 99.9/99.37 | CC | OP491905 |
| FCF21 | Honeycomb |  | *Streptomyces cavourensis* NBRC 13026 | 99.9/99.58 | CC | OP491906 |
| BTF05 | Honeybee | Head | *Streptomyces cavourensis* NBRC 13026 | 99.9/99.64 | CC | OP491907 |
| BBF01 | Honeybee | Cuticle | *Streptomyces cavourensis* NBRC 13026 | 99.9/99.64 | CC | OP491908 |
| BCF13 | Honeybee | Gut | *Streptomyces cavourensis* NBRC 13026 | 99.9/99.64 | HV | OP491909 |
| BCF21 | Honeybee | Gut | *Streptomyces cavourensis* NBRC 13026 | 99.9/99.64 | HV | OP491911 |
| YCF12 | Honeybee | Larvae | *Streptomyces cavourensis* NBRC 13026 | 99.9/99.64 | AIA | OP491912 |
| FCF50 | Honeycomb |  | *Streptomyces cavourensis* NBRC 13026 | 99.9/99.65 | SCA | OP491913 |
| BTF26 | Honeybee | Head | *Streptomyces cavourensis* NBRC 13026 | 99.9/99.71 | AIA | OP491914 |
| BTF27 | Honeybee | Head | *Streptomyces cavourensis* NBRC 13026 | 99.9/99.86 | CC | OP491915 |
| BFF03 | Honeybee | Abdomen | *Streptomyces cavourensis* NBRC 13026 | 99.9/99.86 | SCA | OP491916 |
| YCF15 | Honeybee | Larvae | *Streptomyces cavourensis* NBRC 13026 | 99.9/99.86 | AIA | OP491917 |
| BCF05 | Honeybee | Gut | *Streptomyces cavourensis* NBRC 13026 | 99.9/99.86 | SCA | OP491918 |
| FCF01 | Honeycomb |  | *Streptomyces rubiginosohelvolus* NBRC 12912 | 99.4/99.23 | GS | OP491919 |
| BFF04 | Honeybee | Abdomen | *Streptomyces badius* NRRL B-2567 | 100/99.78 | SCA | OP491920 |
| YCF09 | Honeybee | Larvae | *Streptomyces badius* NRRL B-2567 | 100/100 | R2A | OP491922 |
| YCF11 | Honeybee | Larvae | *Streptomyces badius* NRRL B-2567 | 100/100 | R2A | OP491923 |
| BBF02 | Honeybee | Cuticle | *Streptomyces setonii* NRRL ISP-5322 | 100/99.78 | CC | OP491924 |
| BBF06 | Honeybee | Cuticle | *Streptomyces setonii* NRRL ISP-5322 | 100/99.78 | CC | OP491925 |
| BTF15 | Honeybee | Head | *Streptomyces setonii* NRRL ISP-5322 | 100/99.78 | SCA | OP491926 |
| BCF17 | Honeybee | Gut | *Streptomyces setonii* NRRL ISP-5322 | 100/99.86 | HV | OP491927 |
| BCF24 | Honeybee | Gut | *Streptomyces setonii* NRRL ISP-5322 | 100/99.78 | R2A | OP491928 |
| BCF02 | Honeybee | Gut | *Streptomyces setonii* NRRL ISP-5322 | 100/100 | HV | OP491929 |
| BFF01 | Honeybee | Abdomen | *Streptomyces setonii* NRRL ISP-5322 | 100/100 | SCA | OP491930 |
| BBF03 | Honeybee | Cuticle | *Streptomyces setonii* NRRL ISP-5322 | 100/100 | HV | OP491931 |
| YCF14 | Honeybee | Larvae | *Streptomyces setonii* NRRL ISP-5322 | 100/100 | CC | OP491932 |
| BBF04 | Honeybee | Cuticle | *Streptomyces anulatus* NRRL B-2000 | 100/99.71 | AIA | OP491933 |
| BCF15 | Honeybee | Gut | *Streptomyces anulatus* NRRL B-2000 | 100/99.78 | R2A | OP491934 |
| BCF20 | Honeybee | Gut | *Streptomyces anulatus* NRRL B-2000 | 100/99.93 | R2A | OP491935 |
| BFF07 | Honeybee | Abdomen | *Streptomyces pratensis* ch24 | 94.6/99.93 | CC | OP491936 |
| FCF41 | Honeycomb |  | *Streptomyces pratensis* ch24 | 94.6/100 | CC | OP491937 |
| FCF42 | Honeycomb |  | *Streptomyces pratensis* ch24 | 94.6/100 | CC | OP491938 |
| BTF13 | Honeybee | Head | *Streptomyces pratensis* ch24 | 94.6/100 | SCA | OP491939 |
| YCF04 | Honeybee | Larvae | *Streptomyces pratensis* ch24 | 94.6/100 | R2A | OP491941 |
| YCF05 | Honeybee | Larvae | *Streptomyces pratensis* ch24 | 94.6/100 | R2A | OP491942 |
| BCF19 | Honeybee | Gut | *Streptomyces tanashiensis* LMG 20274 | 100/99.43 | SCA | OP491943 |
| BCF11 | Honeybee | Gut | *Streptomyces tanashiensis* LMG 20274 | 100/99.49 | SCA | OP491944 |
| BCF04 | Honeybee | Gut | *Streptomyces tanashiensis* LMG 20274 | 100/99.64 | SCA | OP491945 |
| YCF18 | Honeybee | Larvae | *Streptomyces tanashiensis* LMG 20274 | 100/99.64 | SCA | OP491946 |
| BTF28 | Honeybee | Head | *Streptomyces tanashiensis* LMG 20274 | 100/99.64 | SCA | OP491947 |
| FCF28 | Honeycomb |  | *Streptomyces tricolor* NBRC 15461 | 99.0/99.37 | R2A | OP491948 |
| FCF52 | Honeycomb |  | *Streptomyces anthocyanicus* NBRC 14892 | 99.9/99.93 | R2A | OP491949 |
| BTF30 | Honeybee | Head | *Streptomyces albidoflavus* DSM 40455 | 99.7/99.50 | CC | OP491950 |
| YCF17 | Honeybee | Larvae | *Streptomyces albidoflavus* DSM 40455 | 99.7/99.57 | CC | OP491951 |
| YCF16 | Honeybee | Larvae | *Streptomyces albidoflavus* DSM 40455 | 99.7/99.64 | SCA | OP491952 |
| YCF07 | Honeybee | Larvae | *Streptomyces albidoflavus* DSM 40455 | 99.7/99.71 | SCA | OP491953 |
| YCF01 | Honeybee | Larvae | *Streptomyces albidoflavus* DSM 40455 | 99.7/99.71 | SCA | OP491954 |

**Table S3.** The relative abundance of OTUs at family level from the phylum Actinobacteria of honeybee gut.

| **Family** | **Relative abundance (%)** |
| --- | --- |
| Bifidobacteriaceae | 97.24 |
| Microbacteriaceae | 0.77 |
| Mycobacteriaceae | 0.35 |
| Micrococcaceae | 0.35 |
| Nocardioidaceae | 0.26 |
| Corynebacteriaceae | 0.19 |
| Nocardiaceae | 0.26 |
| Intrasporangiaceae | 0.18 |
| Streptomycetaceae | 0.10 |
| Frankiaceae | 0.07 |
| Micromonosporaceae | 0.08 |
| Geodermatophilaceae | 0.05 |
| Pseudonocardiaceae | 0.02 |
| Nakamurellaceae | 0.05 |
| Kineosporiaceae | 0.03 |

**Table S4**. The relative abundance of OTUs at family level from the phylum Actinobacteria of honeycomb.

| **Family** | **Relative abundance (%)** |
| --- | --- |
| Nocardiaceae | 12.68 |
| Pseudonocardiaceae | 20.38 |
| Intrasporangiaceae | 10.72 |
| Nocardioidaceae | 12.02 |
| Micrococcaceae | 11.19 |
| Microbacteriaceae | 8.28 |
| Bifidobacteriaceae | 3.77 |
| Micromonosporaceae | 3.64 |
| Brevibacteriaceae | 2.71 |
| Dermabacteraceae | 2.47 |
| Kineosporiaceae | 2.58 |
| Propionibacteriaceae | 1.54 |
| Corynebacteriaceae | 1.72 |
| Cellulomonadaceae | 1.59 |
| Nakamurellaceae | 1.17 |
| Geodermatophilaceae | 1.48 |
| Mycobacteriaceae | 0.88 |
| Dermacoccaceae | 0.49 |
| Streptomycetaceae | 0.26 |
| Nocardiopsaceae | 0.23 |
| Promicromonosporaceae | 0.10 |
| Catenulisporaceae | 0.05 |
| Beutenbergiaceae | 0.05 |

**Table S5.** Antibacterial activity of honeybee-associated actinobacteria against the test pathogens (mm).

| **Isolate code** | ***S. aureus*** | ***M. tetragenus*** | ***E. coli*** | **Psa** |
| --- | --- | --- | --- | --- |
| BTF01 | 8.00±0.00 | 9.00±0.00 | NI | 8.00±0.00 |
| BTF07 | 8.00±0.00 | 7.00±0.00 | NI | NI |
| BTF12 | 8.33±0.47 | 9.50±0.41 | NI | NI |
| FCF02 | 8.00±0.00 | 7.00±0.00 | NI | NI |
| BCF01 | 9.00±0.82 | 7.33±0.47 | NI | 8.67±0.47 |
| FCF23 | 8.00±0.00 | 8.67±0.47 | NI | 15.33±0.47 |
| BTF08 | 7.67±0.62 | 9.67±0.47 | NI | NI |
| FCF40 | 7.00±0.00 | NI | NI | NI |
| FCF44 | 8.33±0.47 | 8.00±0.00 | NI | NI |
| BFF05 | 7.50±0.47 | NI | NI | 11.67±0.47 |
| FCF30 | NI | 7.00±0.00 | NI | NI |
| FCF21 | 7.00 | 9.17±0.24 | NI | 7.33±0.47 |
| BTF05 | 7.50±0.41 | 26.33±2.78 | 9.83±0.85 | 7.00±0.00 |
| BBF01 | 7.00±0.00 | 7.00±0.00 | NI | NI |
| BCF13 | 7.50±0.41 | 8.25±0.25 | NI | 7.33±0.47 |
| BCF21 | 8.00±0.00 | NI | NI | 7.00±0.00 |
| YCF12 | 7.33±0.24 | 8.17±0.24 | 7.00±0.00 | NI |
| FCF50 | 7.00±0.00 | 8.00±0.00 | NI | NI |
| BTF27 | 7.00±0.00 | NI | NI | NI |
| YCF15 | 7.00±0.00 | 7.67±0.47 | 7.00±0.00 | NI |
| BCF05 | 7.67±0.47 | 8.83±0.24 | 8.67±0.24 | NI |
| FCF01 | 23.00±1.41 | 15.00±0.00 | NI | 13.33±0.47 |
| BFF04 | 12.00±0.00 | 11.50±0.41 | NI | 14.00±0.82 |
| YCF09 | 7.33±0.24 | 7.17±0.24 | 7.33±0.47 | 7.00±0.00 |
| YCF11 | 8.17±0.24 | NI | 8.00±0.00 | 10.00±0.00 |
| BBF02 | 7.00±0.00 | NI | NI | 8.00±0.00 |
| BTF15 | 7.17±0.24 | 27.33±0.62 | 10.00±0.00 | NI |
| BCF17 | NI | NI | NI | NI |
| BCF24 | NI | NI | NI | NI |
| BCF02 | 7.83±0.24 | 39.33±0.94 | 11.33±0.62 | 12.67±0.47 |
| BBF03 | NI | NI | NI | NI |
| YCF14 | NI | NI | NI | NI |
| BBF04 | NI | NI | NI | 8.00±0.00 |
| BCF15 | 7.67±0.47 | 13.00±1.41 | NI | NI |
| FCF41 | NI | 7.00±0.00 | NI | 8.00±0.00 |
| BTF13 | 7.00±0.00 | NI | NI | 7.67±0.47 |
| YCF04 | NI | NI | NI | NI |
| YCF05 | NI | NI | NI | 8.33±0.47 |
| BCF11 | NI | NI | NI | NI |
| BCF04 | 7.33±0.47 | 10.33±0.47 | NI | 13.67±0.47 |
| YCF18 | NI | NI | NI | NI |
| BTF28 | 7.00±0.00 | NI | NI | NI |
| FCF28 | 7.00±0.00 | 8.33±0.47 | NI | NI |
| FCF52 | NI | NI | NI | NI |
| BTF30 | NI | NI | NI | NI |
| YCF17 | 7.33±0.24 | NI | NI | NI |
| YCF16 | NI | NI | NI | 7.33±0.47 |
| YCF07 | NI | NI | NI | NI |
| YCF01 | NI | NI | NI | NI |
| Gentamicin sulfate^a^ | 21.67±2.87 | 37.67±2.05 | 20.00±2.83 | 22.67±0.47 |

^a^Gentamicin sulfate as the positive control of pathogenic bacteria; results are presented as the mean ± standard; “NI” means not inhibited; the concentration for the test is 50 µg/filter paper.


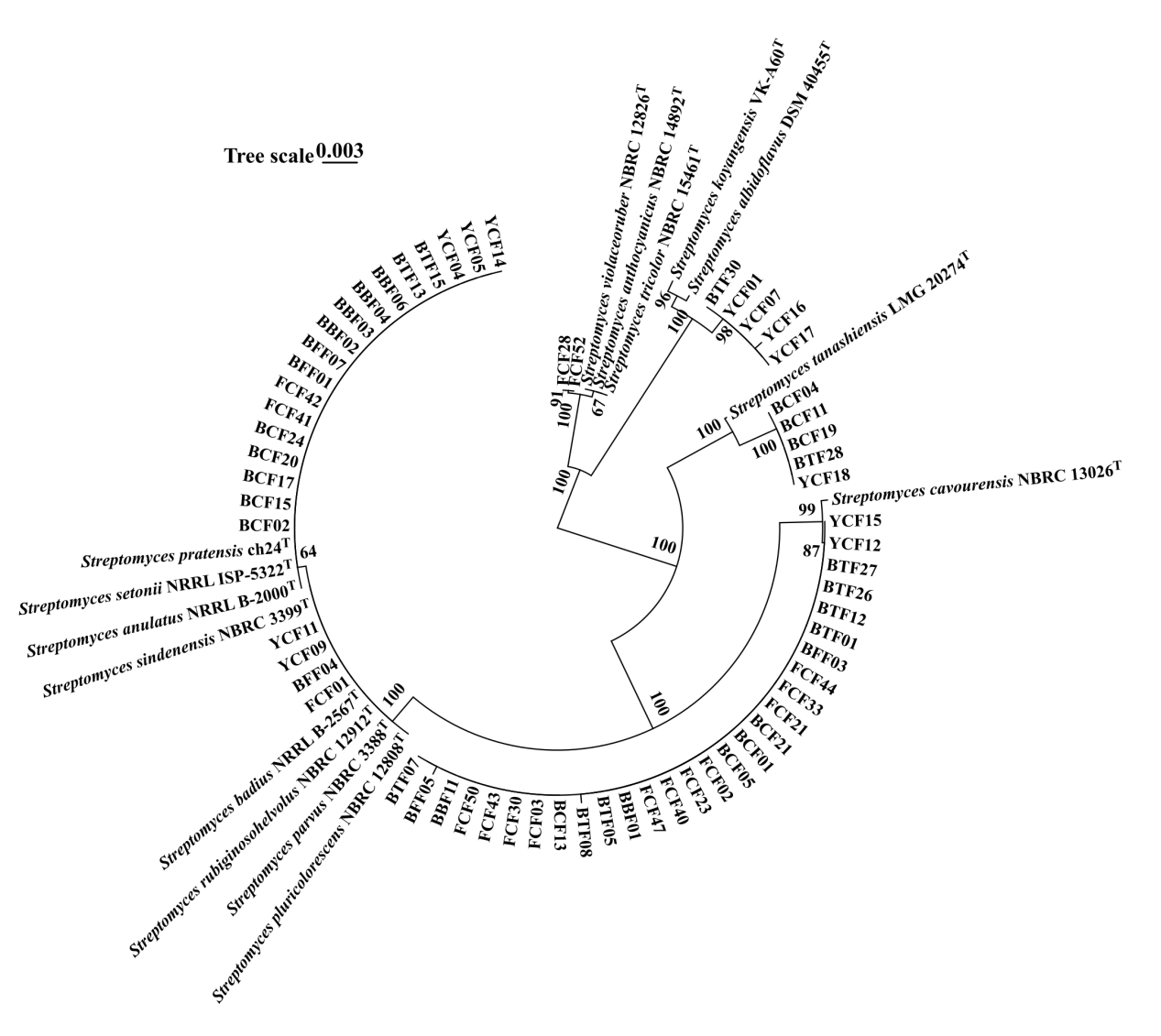


**Figure S1**. Neighbor-joining phylogenetic tree of 16S rRNA gene sequences from honeybee-associated actinobacteria and their phylogenetic neighbors. Numbers at nodes are bootstrap values (percentages of 1000 replications); only values >50% are shown.


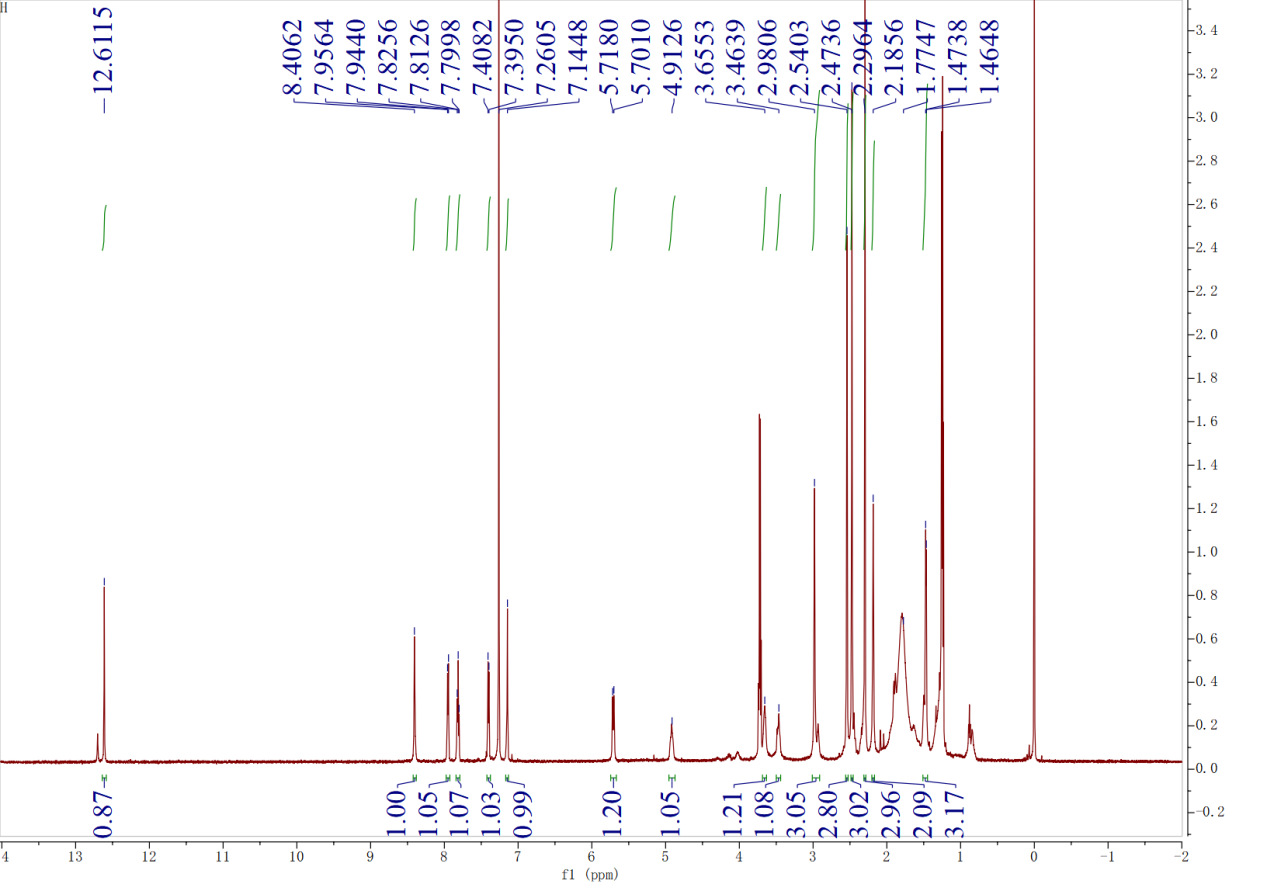


**Figure S2.** The ^1^H NMR spectrum of compound **1a** in CDCl_3_ (600 MHz).


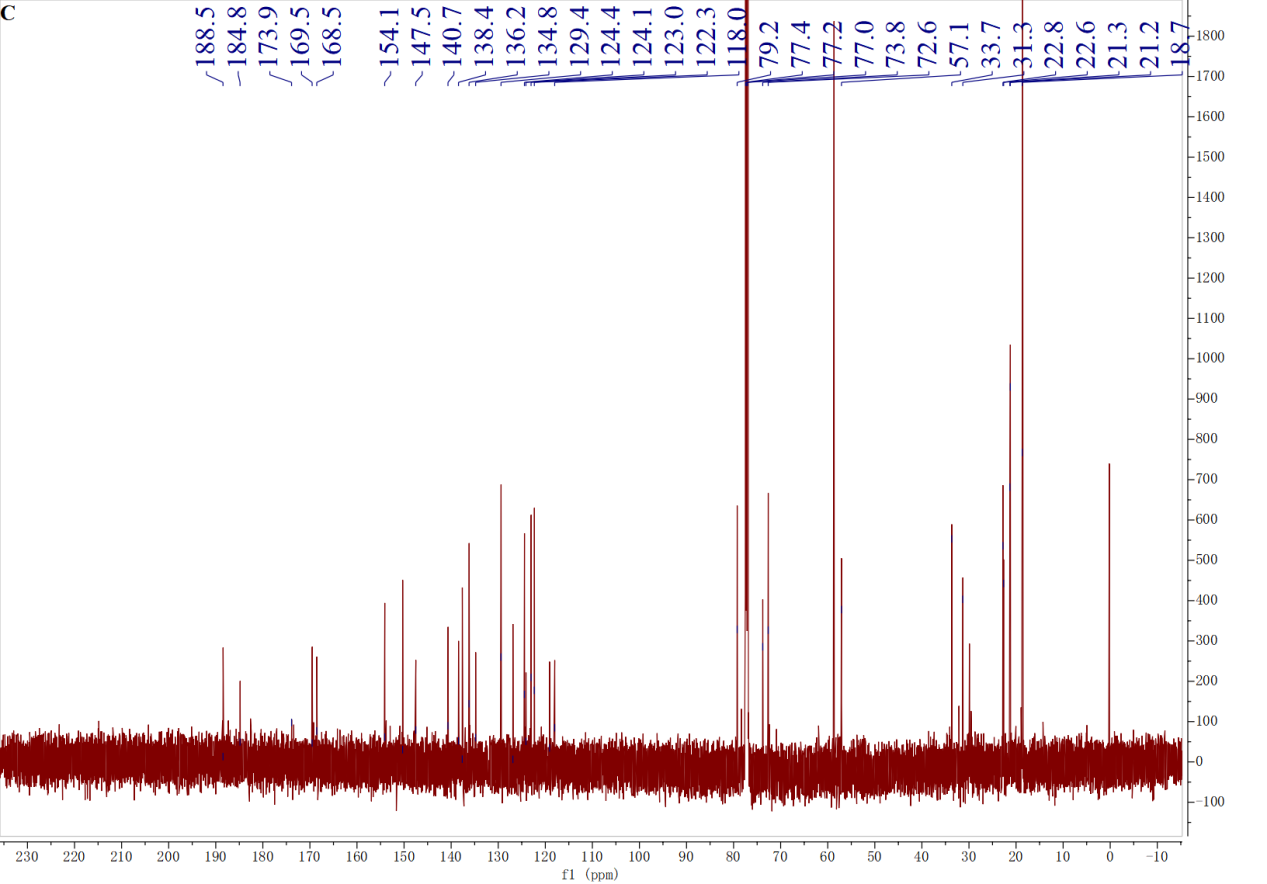


**Figure S3.** The ^13^C NMR spectrum of compound **1a** in CDCl_3_ (150 MHz).


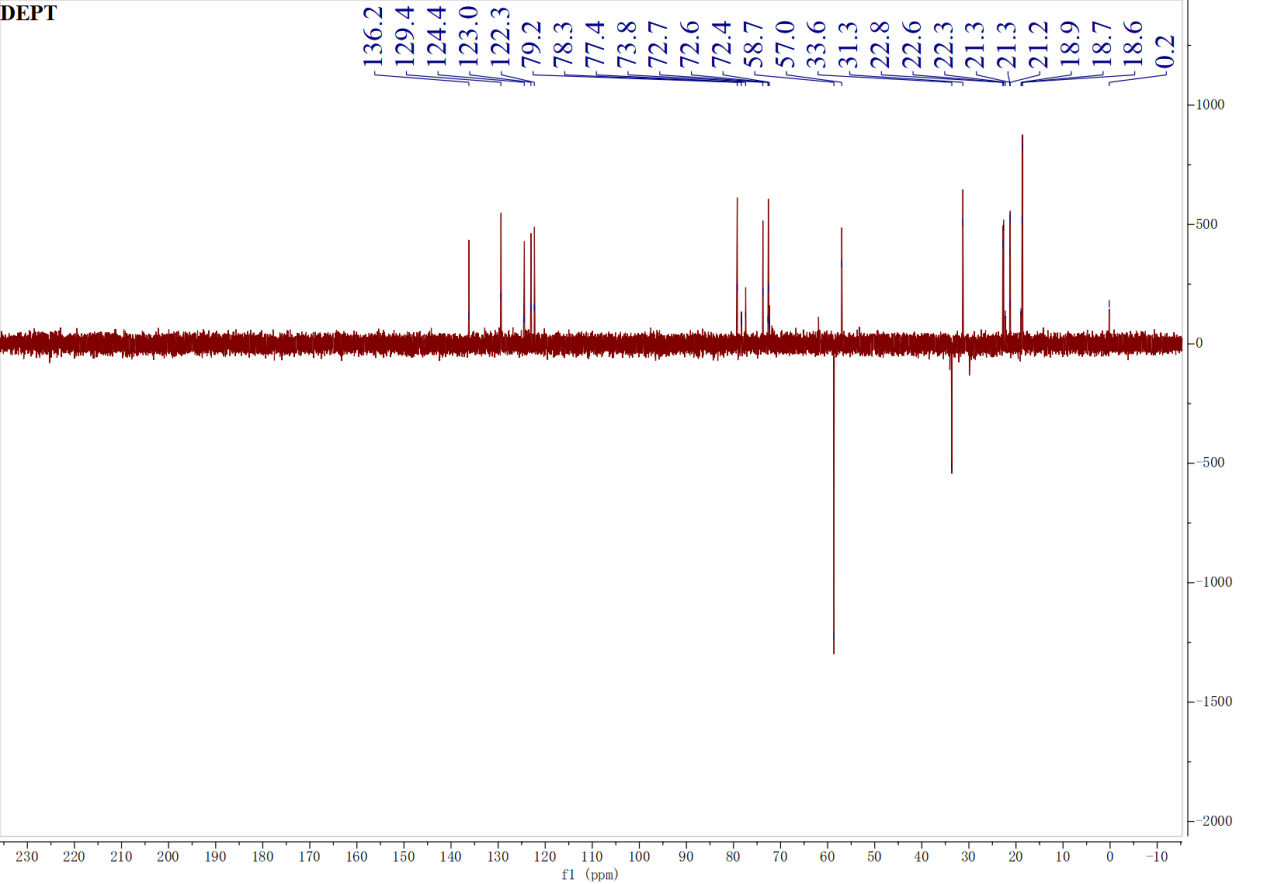


**Figure S4.** The DEPT spectrum of compound **1a** in CDCl_3_ (150 MHz).


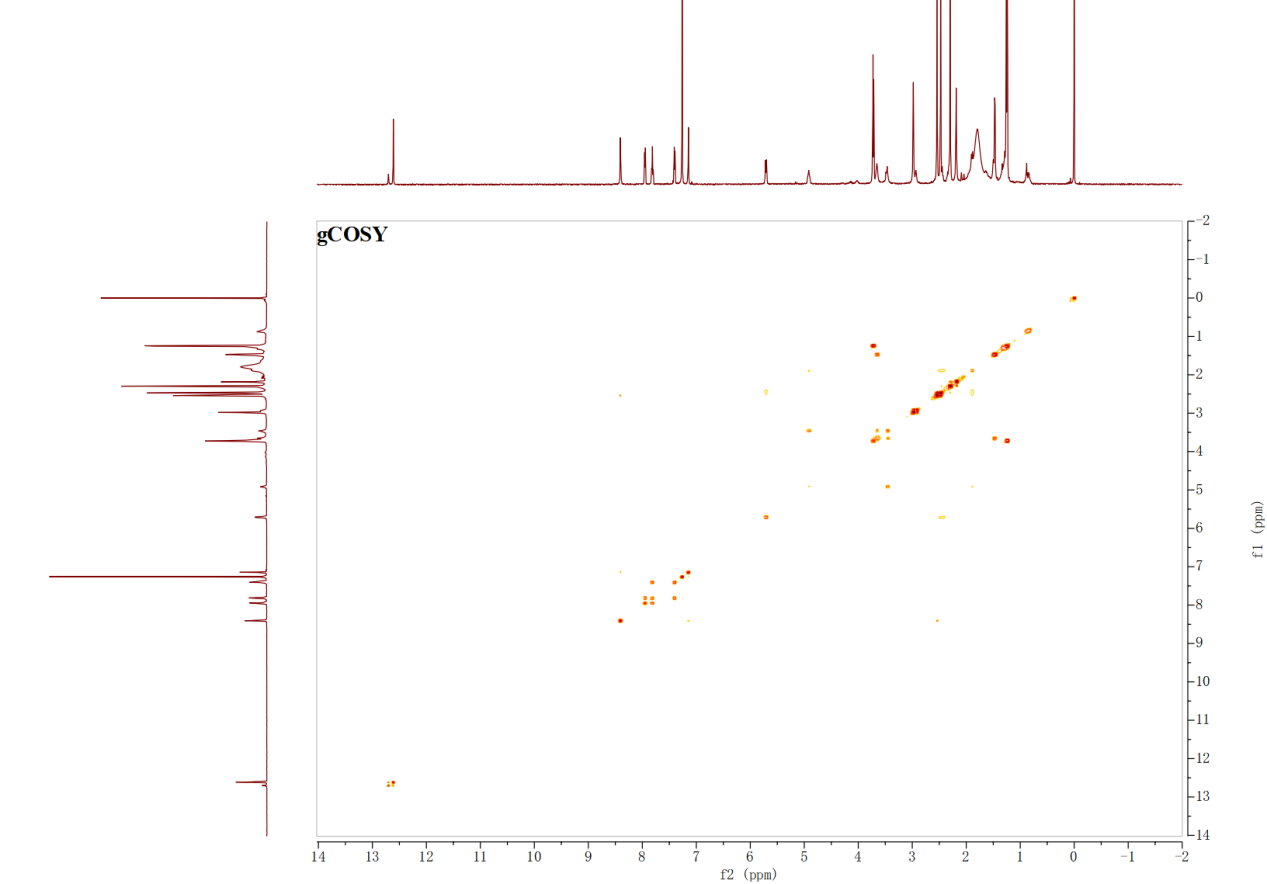


**Figure S5.** The ^1^H-^1^H COSY spectrum of compound **1a** in CDCl_3_ (600 MHz).


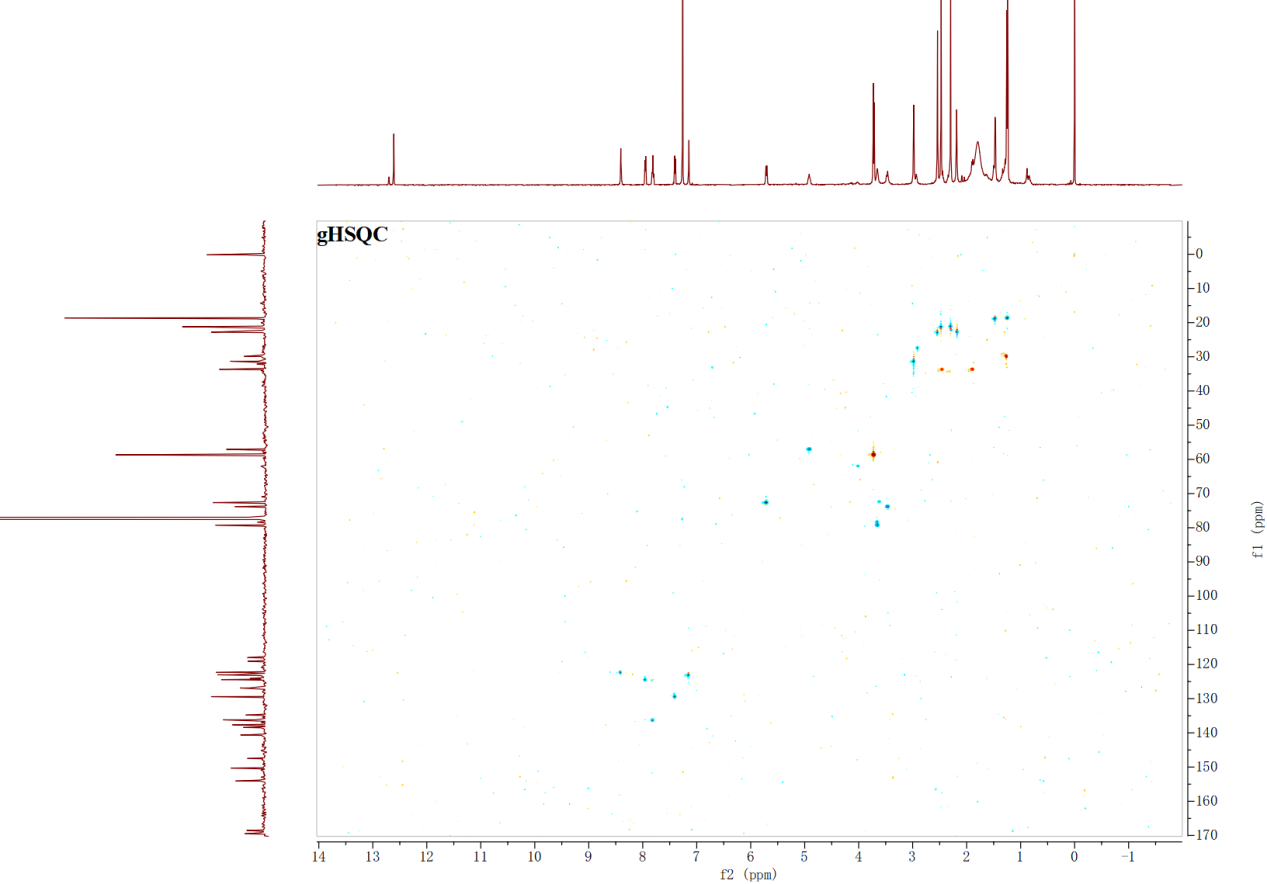


**Figure S6.** The HSQC spectrum of compound **1a** in CDCl_3_ (600 MHz).


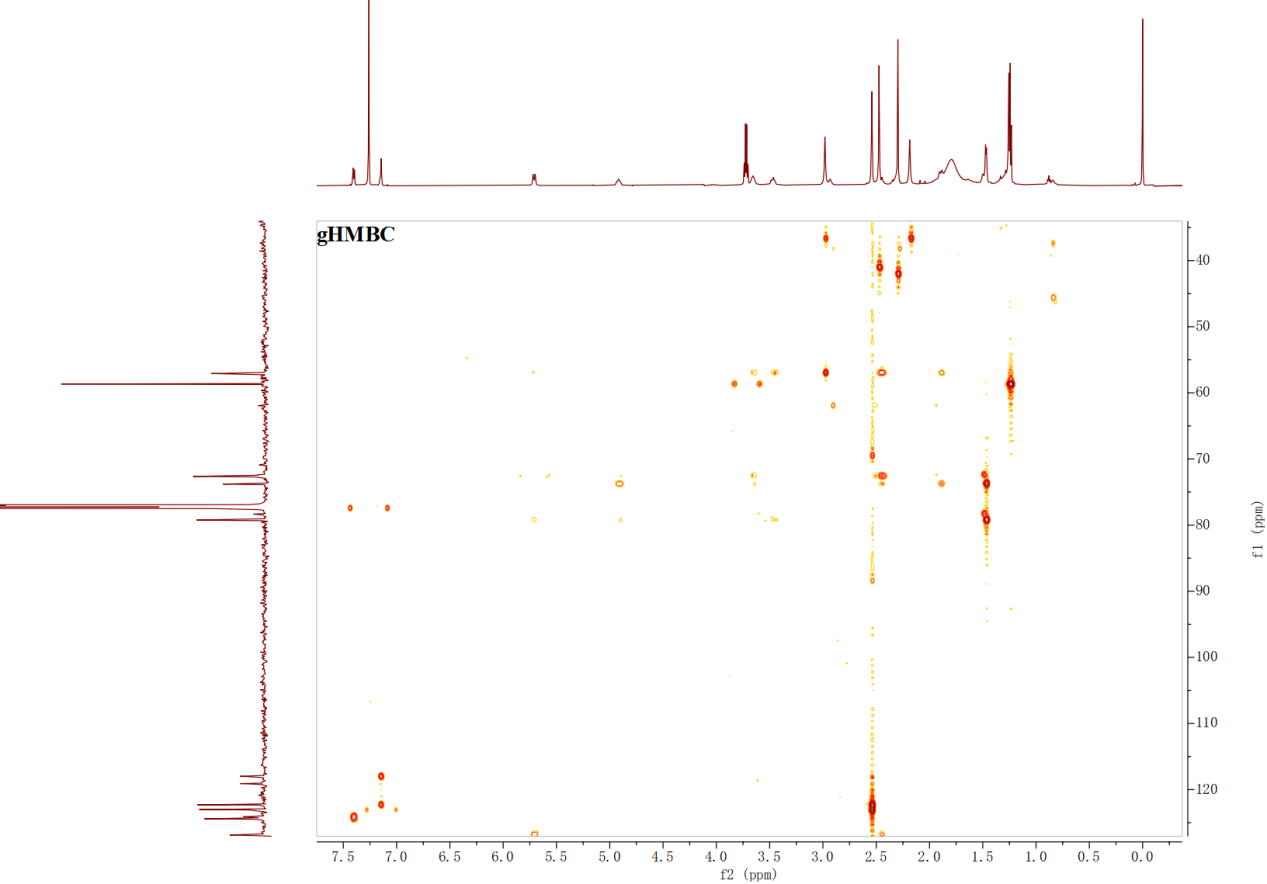


**Figure S7.** The HMBC spectrum of compound **1a** in CDCl_3_ (600 MHz).


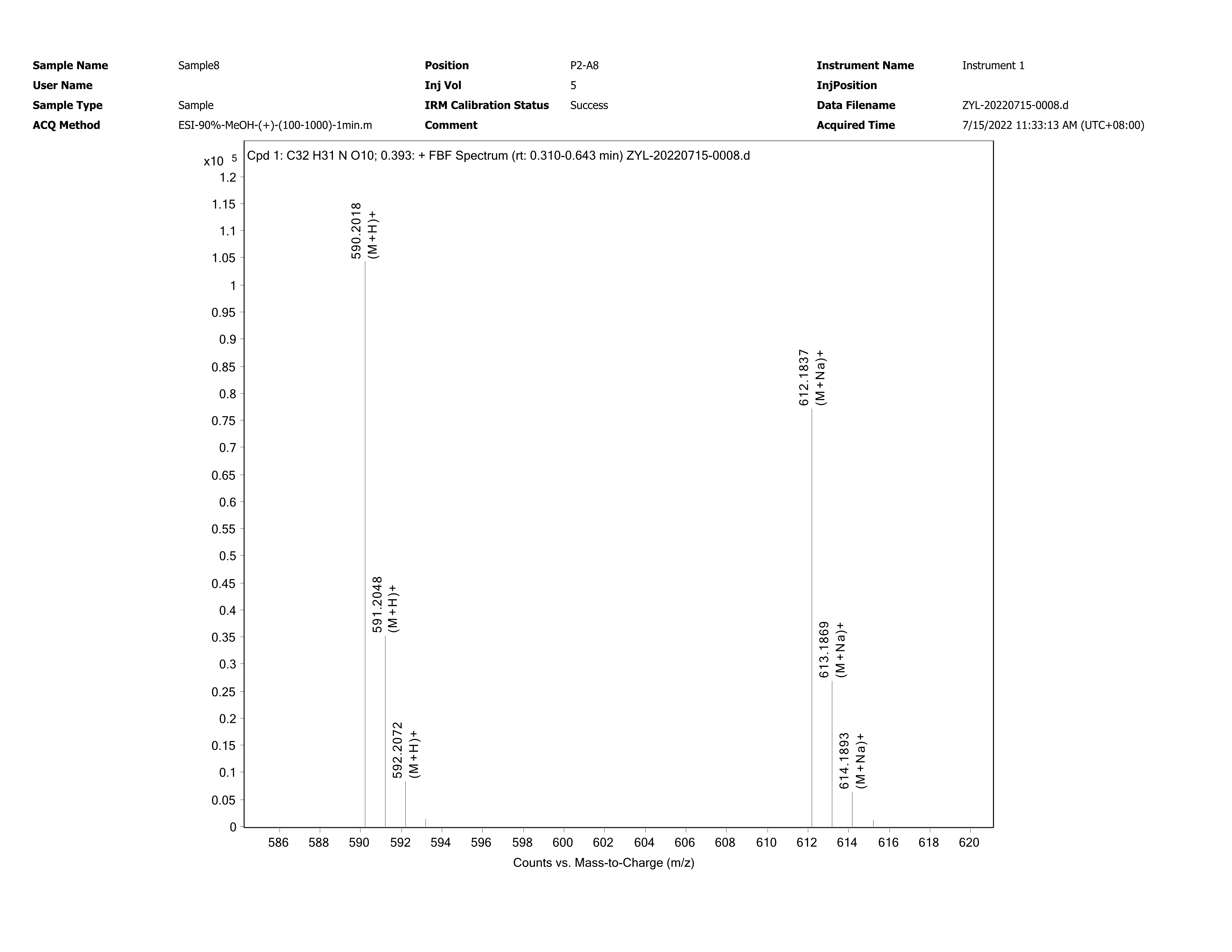


**Figure S8.** The ESI^+^ spectrum of compound **1a**.


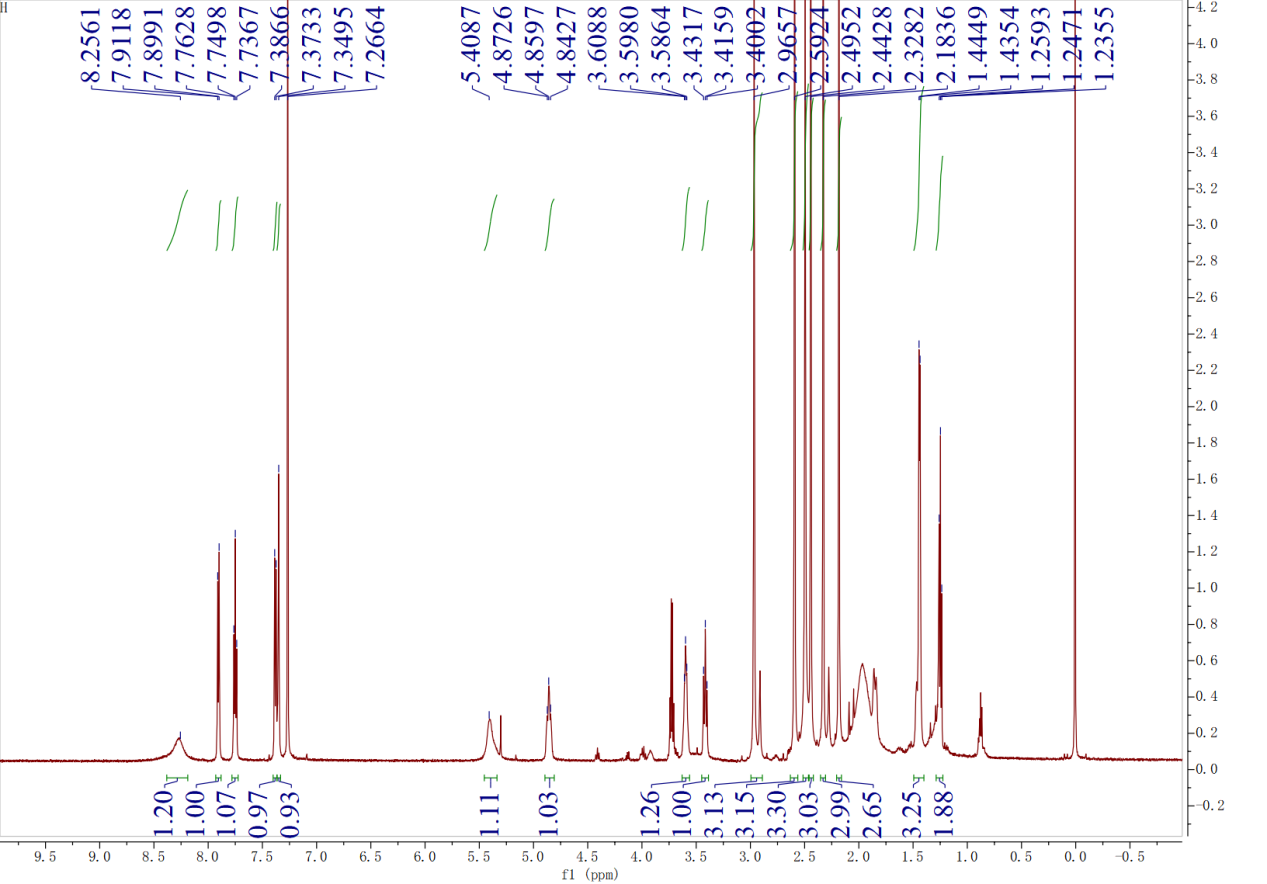


**Figure S9.** The ^1^H NMR spectrum of compound **1b** in CDCl_3_ (600 MHz).

**
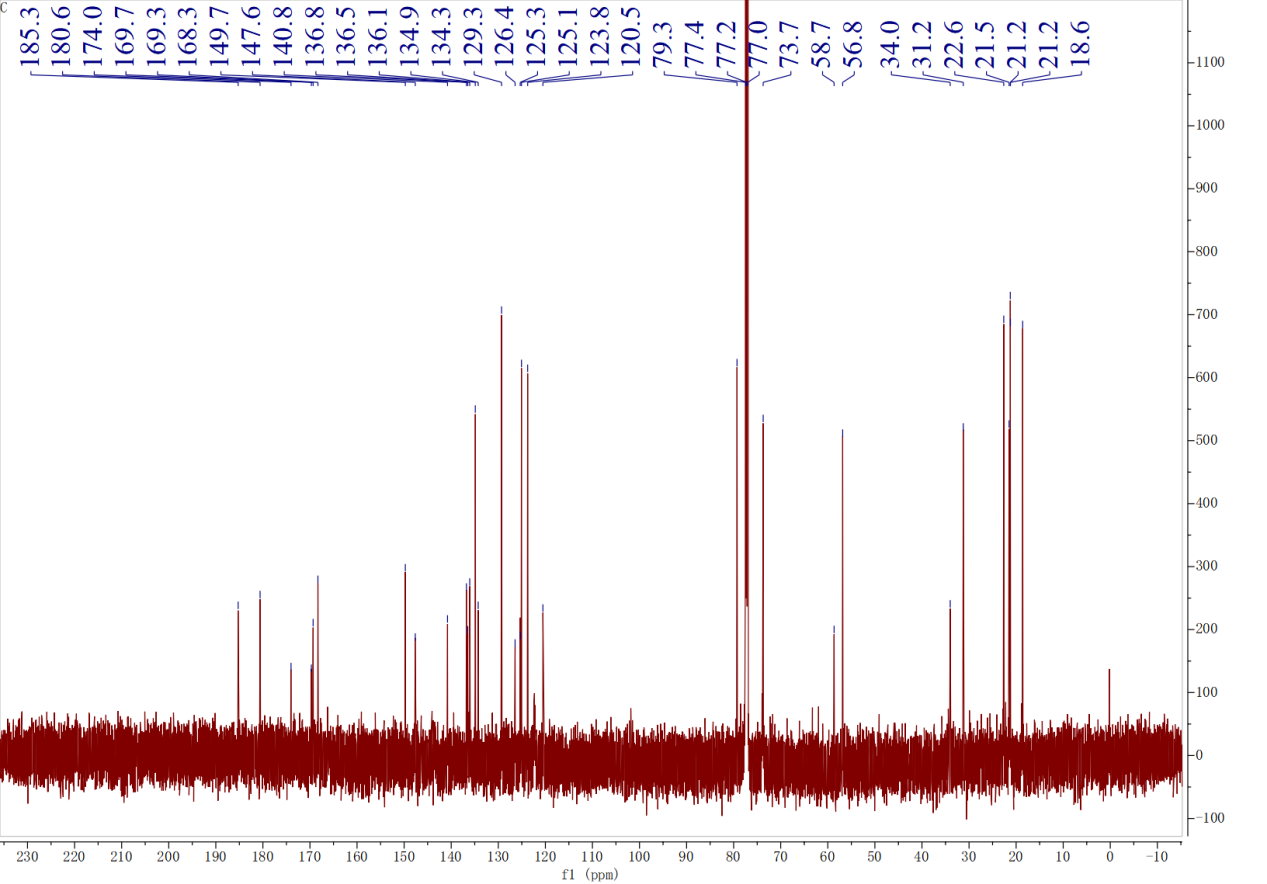
**

**Figure S10.** The ^13^C NMR spectrum of compound **1b** in CDCl_3_ (150 MHz).


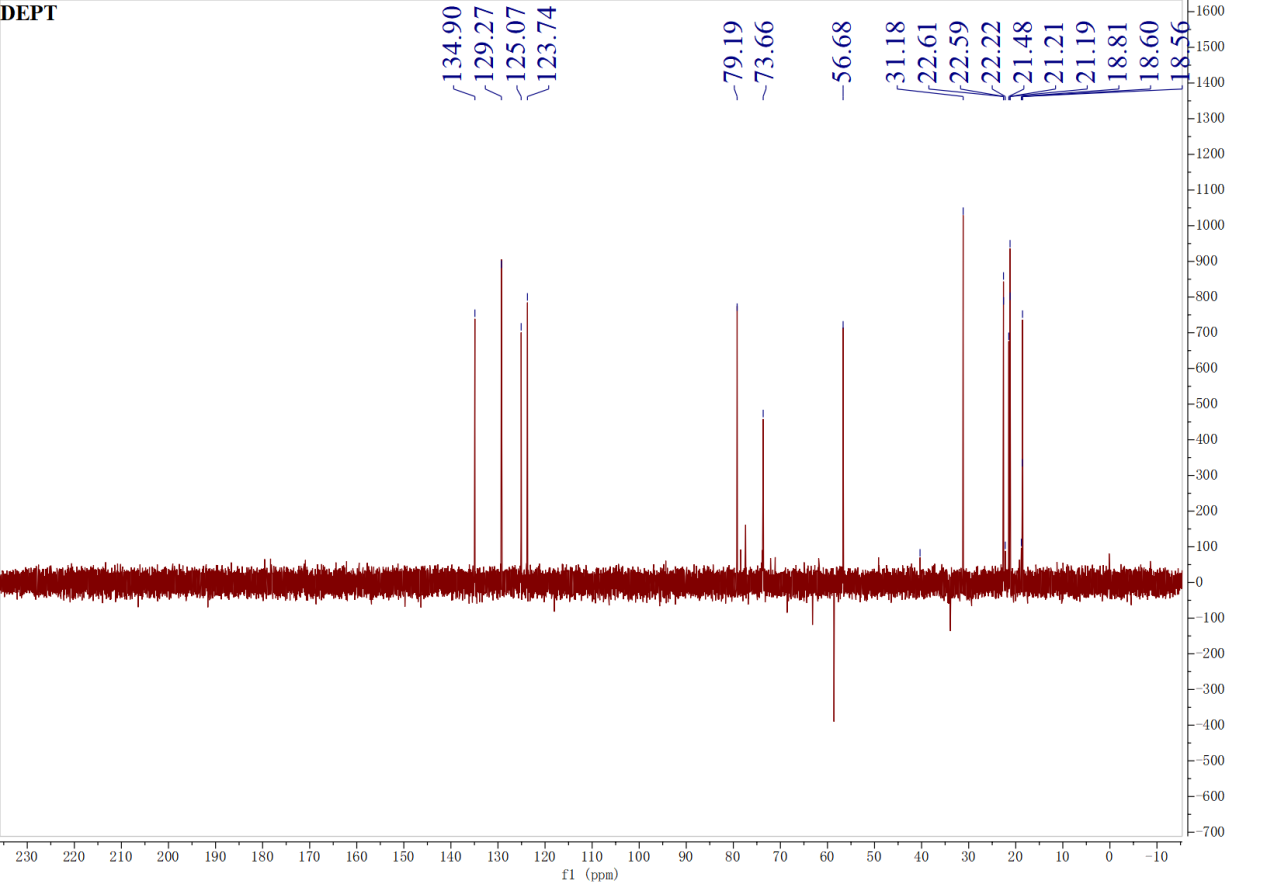


**Figure S11.** The DEPT spectrum of compound **1b** in CDCl_3_ (150 MHz).

**
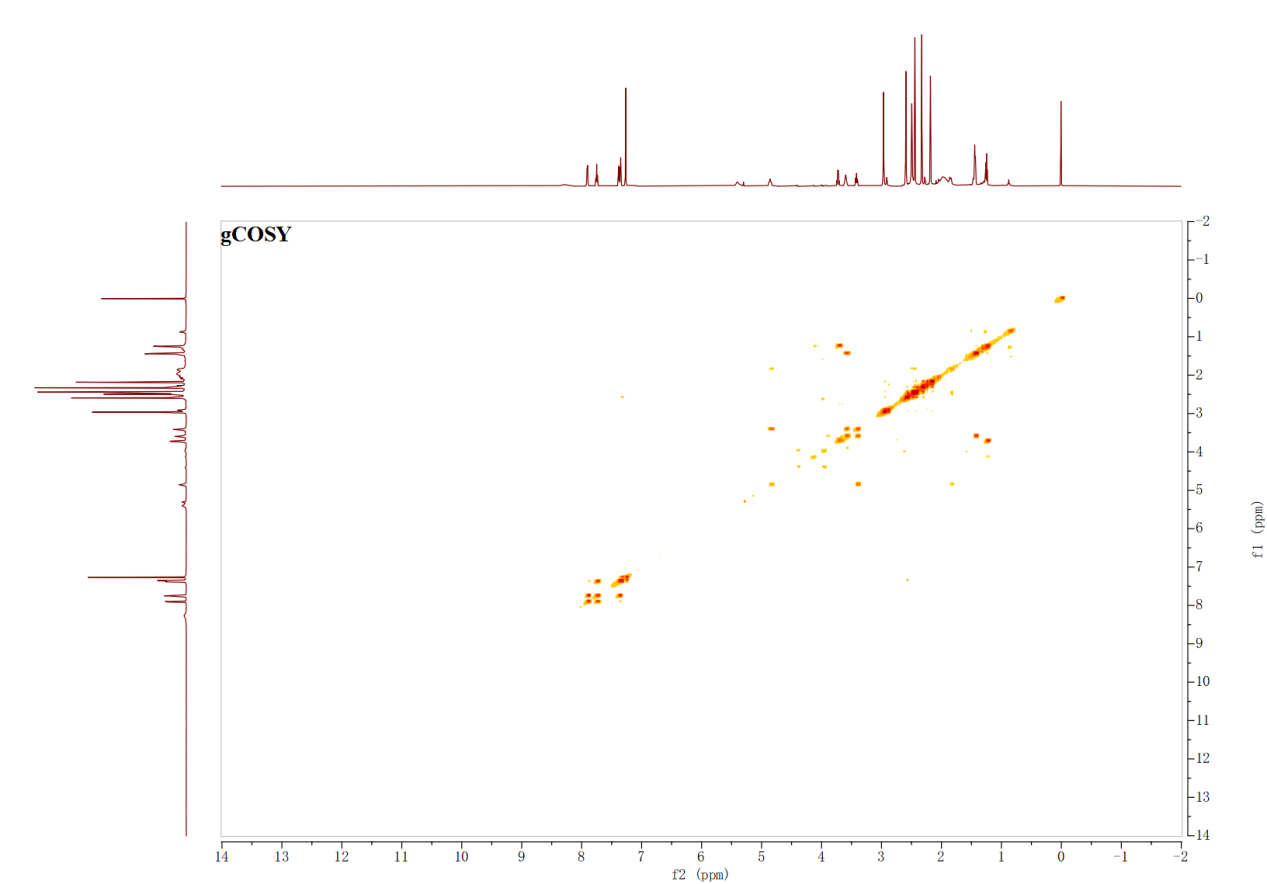
**

**Figure S12.** The ^1^H-^1^H COSY spectrum of compound **1b** in CDCl_3_ (600 MHz).


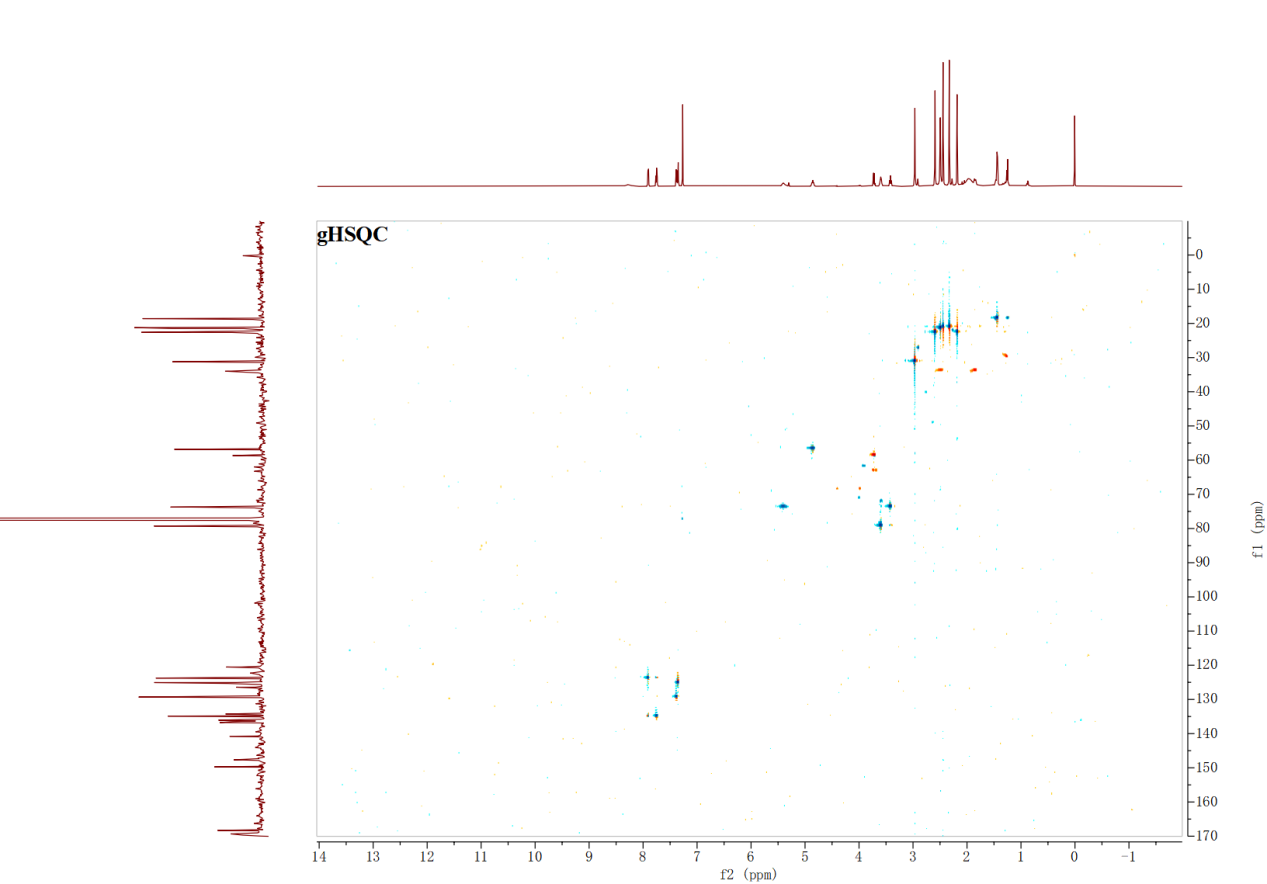


**Figure S13.** The HSQC spectrum of compound **1b** in CDCl_3_ (600 MHz).

**
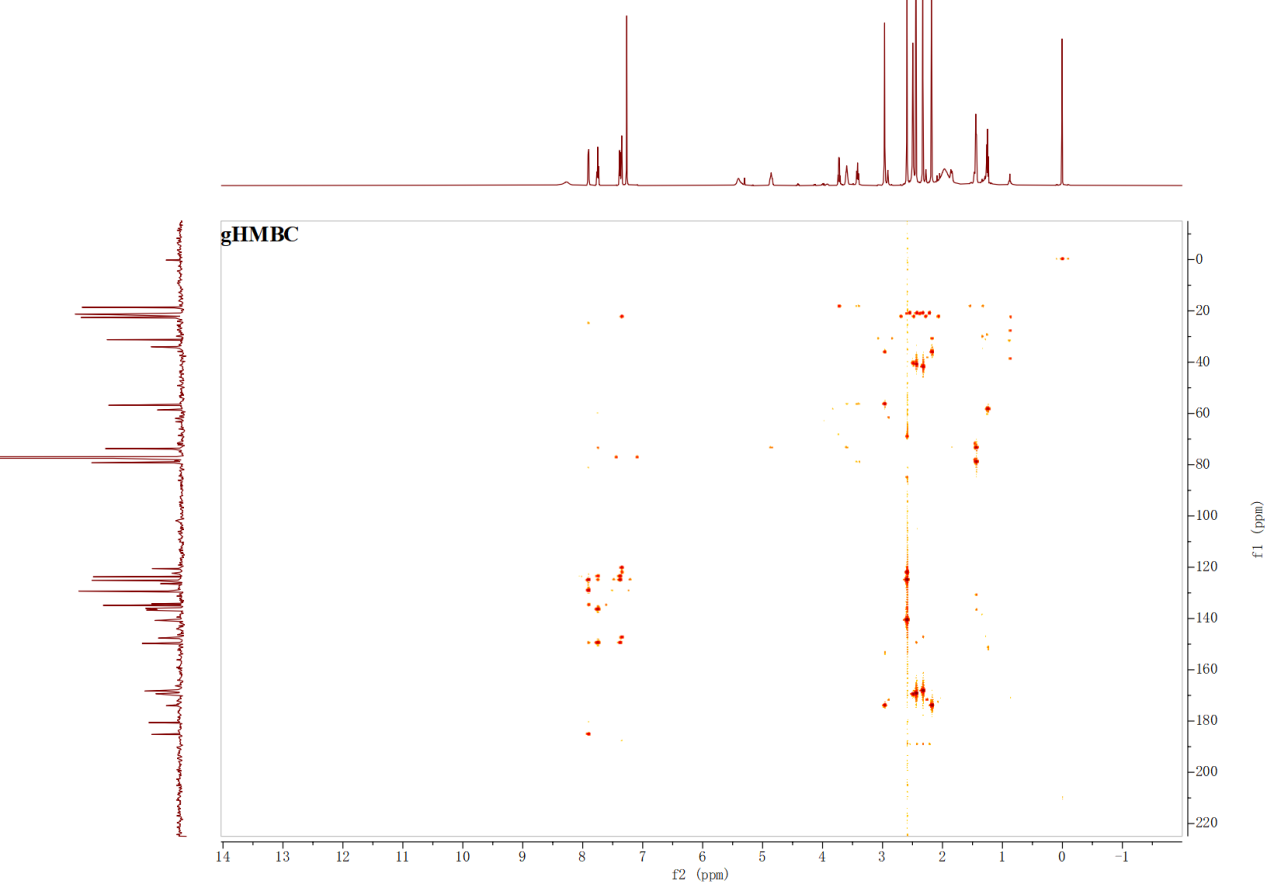
**

**Figure S14.** The HMBC spectrum of compound **1b** in CDCl_3_ (600 MHz).


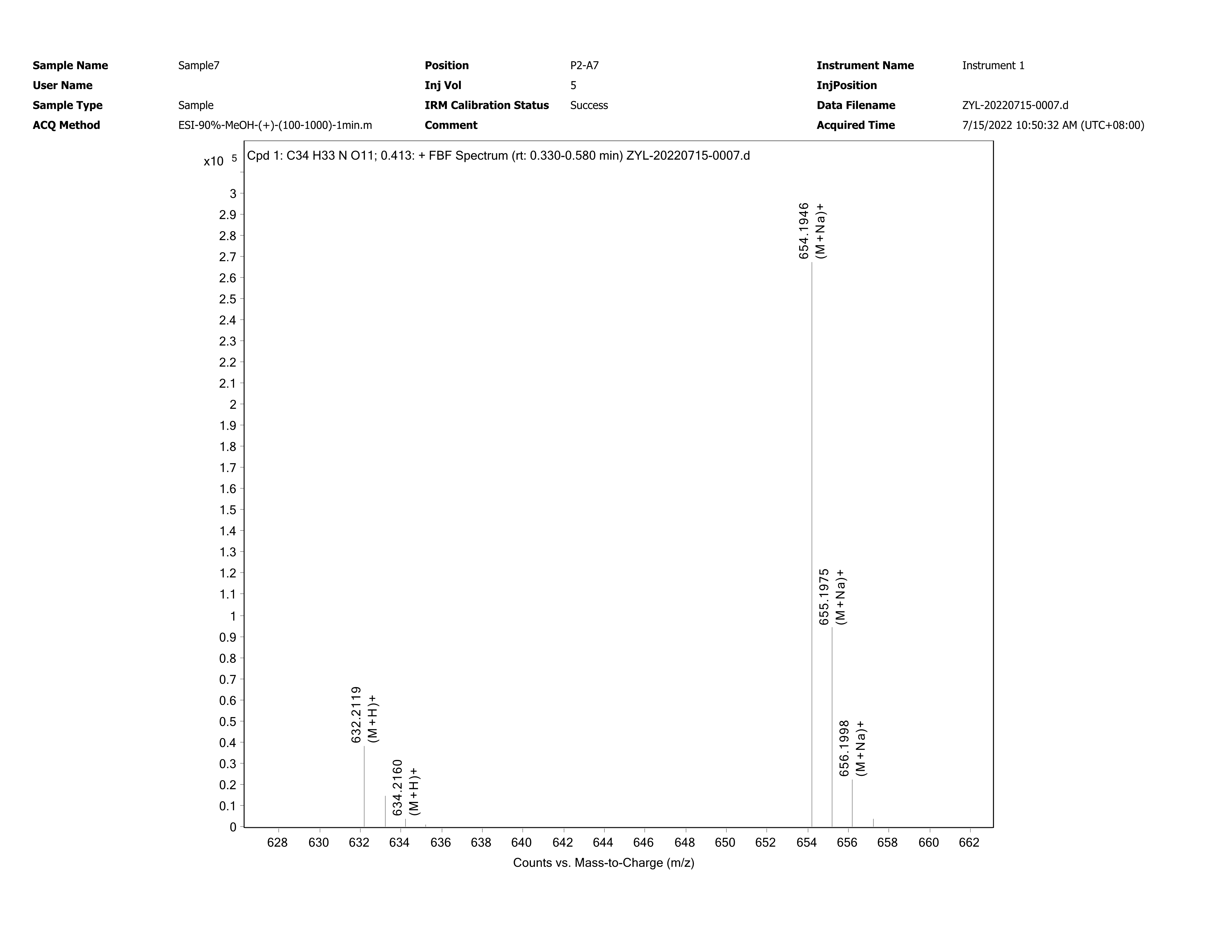


**Figure S15.** The ESI^+^ spectrum of compound **1b**.
